# The catalytic inactivation of the N-half of human hexokinase 2 and structural and biochemical characterization of its mitochondrial conformation

**DOI:** 10.1042/BSR20171666

**Published:** 2018-02-21

**Authors:** Mir Hussain Nawaz, Juliana C. Ferreira, Lyudmila Nedyalkova, Haizhong Zhu, César Carrasco-López, Serdal Kirmizialtin, Wael M. Rabeh

**Affiliations:** 1 Science Division, New York University Abu Dhabi, P.O. Box 129188, Abu Dhabi, United Arab Emirates; 2 Structural Genomics Consortium, University of Toronto, Toronto, Ontario M5G 1L7, Canada

**Keywords:** Apoptosis, Cancer Metabolism, Hexokinase 2, Mitochondria

## Abstract

The high proliferation rate of tumor cells demands high energy and metabolites that are sustained by a high glycolytic flux known as the ‘Warburg effect’. The activation and further metabolism of glucose is initiated by hexokinase, a focal point of metabolic regulation. The human hexokinase 2 (HK2) is overexpressed in all aggressive tumors and predominantly found on the outer mitochondrial membrane, where interactions through its N-terminus initiates and maintains tumorigenesis. Here, we report the structure of HK2 in complex with glucose and glucose-6-phosphate (G6P). Structural and biochemical characterization of the mitochondrial conformation reveals higher conformational stability and slow protein unfolding rate (*k*_u_) compared with the cytosolic conformation. Despite the active site similarity of all human hexokinases, the N-domain of HK2 is catalytically active but not in hexokinase 1 and 3. Helix-α_13_ that protrudes out of the N-domain to link it to the C-domain of HK2 is found to be important in maintaining the catalytic activity of the N-half. In addition, the N-domain of HK2 regulates the stability of the whole enzyme in contrast with the C-domain. Glucose binding enhanced the stability of the wild-type (WT) enzyme and the single mutant D657A of the C-domain, but it did not increase the stability of the D209A mutant of the N-domain. The interaction of HK2 with the mitochondria through its N-half is proposed to facilitate higher stability on the mitochondria. The identification of structural and biochemical differences between HK2 and other human hexokinase isozymes could potentially be used in the development of new anticancer therapies.

## Introduction

A high rate of glucose metabolism is essential to support the rapid growth of different types of tumors that utilize aerobic glycolysis rather than oxidative phosphorylation, a phenomenon known as the Warburg effect [1–3]. The high demand for glucose is necessary for energy production and the synthesis of building blocks required for the rapidly proliferating cancer cells [3]. Hexokinase, the first enzyme of the glycolytic pathway, catalyzes the irreversible rate-limiting phosphorylation of glucose to glucose-6-phosphate (G6P). The role of hexokinase is essential not only for the intracellular retention of glucose, but also for its activation and further metabolism [4]. The hexokinase reaction is also a focal point for the regulation of glucose metabolism and other biosynthetic pathways that require glycolytic intermediates for their function, making hexokinase an important target for the regulation of tumor growth and metastasis.

In mammals, four hexokinase isozymes have been characterized, including HK1, HK2, and HK3 with two domains (~100 kDa) that are structurally similar to the single domain HK4 (52 kDa), also known as glucokinase [5]. The elevated rates of glucose metabolism, tumor progression, and the high patient mortality has been predominantly associated with the overexpression of HK2 in different cancers [6]. This phenotype has been utilized clinically for the diagnosis of cancer by Positron Emission Tomography (PET) scan [7]. In the liver, for example glucokinase is the predominantly expressed enzyme in normal adult hepatocytes. Tumorigenesis is initiated in the liver after a switch in gene expression from glucokinase (HK4) to high levels of HK2 [8,9]. Furthermore, the up-regulation of HK2 has been shown to facilitate the chemotherapy resistance and metastasis in different cancer cells [10].

HK2 is predominantly co-localized on the outer mitochondrial membrane (OMM), where its translocation is facilitated by an increase in the cellular glucose concentration [11–13]. The mitochondrial translocation of HK2 not only gives it preferential access to ATP required for its function and relieves feedback inhibition by G6P, but most importantly, suppresses apoptosis [14,15]. HK2 localization on the OMM prevents the formation of the mitochondrial permeability transition pore, which halts apoptosis and increases tumor cell viability and resistance to chemotherapeutic agents [10]. Conversely, HK2 dissociation promotes mitochondrial injury and decreases tumor growth, which makes it an attractive target for the development of anticancer therapies [15].

Here, we determined the crystal structure of human HK2 in complex with glucose and G6P with structural fold similar to other mammalian hexokinases [16–23]. Mitochondrial interaction is facilitated by the mitochondrial-binding peptide (MBP), the first helix on the N-terminus of HK2. The mitochondrial conformation was generated after deleting the MBP (Δ16-HK2) and was found to possess higher conformational stability in comparison with the full-length (FL) HK2 variant.

A total loss of the catalytic activity in the N-half of HK2 was observed after altering the size of the linker helix-α_13_ that connects the N- to C-domain. The catalytic activity of the N-domain was proportional to the length of the linker helix-α_13_, where an N-domain variant with a short helix-α_13_ was catalytically inactivate. Computational modeling showed the linker helix-α_13_ to remotely modulate glucose binding by controlling the grove width of the N-domain active site. These findings can explain the inactivation of the N-half of HK1 with similar active site residues to HK2 but different conformation of its linker helix.

The catalytic residue of the N-half, but not the C-half, was found to reduce the extent of glucose stabilization on the whole enzyme. The addition of glucose to the single mutant D209A in the N-domain did not stabilize the whole enzyme, but increased the stability of the wild-type (WT) and D657A mutant enzymes. These results indicate the importance of the N-domain in controlling the stability of the whole enzyme in the addition to its role in binding HK2 to the mitochondria.

## Experimental procedures

### Materials

All chemicals obtained were from Sigma–Aldrich (Germany). The bacterial culture media, buffers, and supplements were purchased from Invitrogen. The synthesis of oligonucleotide DNA primers and DNA sequencing were done by Genscript Inc (Piscataway, NJ). Restriction enzymes were purchased from NEB (Beverly, MA). Ni Sepharose 6 Fast Flow was purchased from GE Healthcare (Sweden). The PCR and restriction digest products were purified using the QIAquick PCR purification kit (Qiagen). KOD DNA polymerase and dNTP mix were from EMD Millipore (Danvers, MA).

### Plasmid construction and protein purification

Different constructs of HK2 were expressed in *E. coli* using Champion pET SUMO expression system (Thermo Fisher Scientific). Point mutations were introduced using the QuickChange™ site-directed mutagenesis kit (Agilent). HK2 expression was induced by 0.2 mM IPTG and the cells were harvested by centrifugation at 8000 rpm for 10 min. The cell pellet was resuspended in 100 ml lysis buffer (100 mM Tris, pH 7.5, 150 mM NaCl, 5% glycerol, 5 mM imidazole) containing protease inhibitor cocktail (Sigma). Cells were lysed by sonication at 20% amplitude for 5 min, then centrifuged at 16000 rpm for 30 min at 4°C. The supernatant was loaded on to ProBond™ Nickel-Chelating Resin (Life Technologies) that was pre-equilibrated with lysis buffer. The column was washed, then eluted with lysis buffer containing 30 and 250 mM imidazole, respectively. The His_6_ tag was cleaved with thrombin protease overnight on ice then loaded on to a HiLoad Superdex 200 column using AKTA FPLC system (GE Life Sciences). Fractions containing HK2 protein were combined and concentrated to ~5 mg/ml in buffer containing 150 mM NaCl, 0.5 mM TCEP, 5% glycerol, and 10 mM HEPES, pH 7.8. The protein samples were analyzed using SDS/PAGE, and the purity of the different HK2 variants was determined from the bands on the Coomassie stained gels using densitometry on ImageJ software [25].

### Crystallization and structural determination

Crystals of Δ16-HK2 were grown by sitting-drop vapor diffusion in a mixture of 16% PEG 3350, 0.2 M sodium malonate, 0.1 M Bis-Tris propane (BTP) pH 8.5, 10% ethylene glycol, and 1 mM DTT at 18°C. Crystals were cryoprotected in a 50:50 mixture of Paratone-N and mineral oil before flash cooling in liquid nitrogen. Diffraction data were collected on Rigaku FR-E with Rigaku RAXIS image plate at a wavelength of 1.5418 Å and data were processed with the HKL2000 suite (Supplementary Table S1) [36]. The crystal structure was solved by the molecular replacement method using Phaser in the CCP4 program suite with human hexokinase 1 (PDB code: 1HKB) as the structural model [18]. The structure was refined with REFMAC5. The treatment for B-factors is isotropic and the dimer is in the asymmetric unit. The model was deposited with PDB code: 2NZT. Figures were prepared with the PyMOL Molecular Graphics System, version 1.8, Schrödinger LLC.

### Computational modeling of the HK2 structure and dynamics

Since HK2 did not crystallize in the absence of substrates, the open state was modeled using the closed state of HK2 (PDB code: 2NZT). Computational modeling approach was used to generate the open state using HK4 (PDB code: 1V4T) as a template using MODELLER [19,37]. Keeping the open state model as the target state, a targetted molecular dynamics (TMD) simulation protocol implemented in Gromacs 4.05 was used with Structure-based Model for biomolecules (SMOG) potential [38–40] to obtain the open state. SMOG potential allowed rapid sampling of the conformational space while keeping the secondary structure stoichiometry intact during the closed-to-open transition. Structure-based potentials have been utilized heavily to study biomolecular processes including the mechanism of conformational transitions of large biomolecular complexes [41]. Here, we used it for the first time to obtain the open state of HK2 based on the initial guess from homology modeling. A homodimer was constructed and energy minimized for both end states. A TMD simulation of 250000 steps in temperature 40K that is kept constant using Velocity Verlet scheme [42]. Stochastic dynamics integrator with a time step of 0.001 ps and a friction coefficient of 0.1 amu/ps was used to integrate the equations of motion. For details on the conversion to real values, see [39]. Non-bonded interactions were treated with 15 Å cutoff. The trajectory created by TMD was later used as the initial guess for minimum energy pathway of open-to-close transition.

The transition pathway from closed-to-open states was constructed from 96 configurations equally spaced from TMD trajectory. The pathway was then refined to minimum energy path using SDP methodology implemented in MOIL [26,43] this time using classical molecular mechanics forcefield. Generalized Born Surface Area (GBSA) model and OPLSAA force field was used to account for the interactions of glucose with HK2 [44,45]. ATP parameters and path sampling protocol were adopted from our previous work [46]. In SDP approach given the two end configurations, *R*_C_ and *R*_O_, a discrete set of coordinates, *R_i_* where index i =2, 3,...,N−1, were established between the two end states and the functional $S[R(l)]\;=\;\int_{R_{C}}^{R_{O}}\;dl\;\sqrt{\nabla U^{t}\nabla U}$ is optimized. Here, *R*(*l*) is the coordinate vector as a function of arc-length *l*, and ∇*U*(*R*(*l*)) is the gradient of the potential energy. The minimum energy path from closed to open states is shown in Supplementary Movie S1.

MD was conducted in explicit water and ions using Gromacs 4.05 suite of programs [40] including only N-half. As in the previous section, OPLSAA force field was used for glucose and protein [47], and ATP parameters were adopted from [46]. Water was modeled using SPCE [48]. Smith and Dang [49] parameters were used to model K^+^ and Cl^−^, and for Mg^2+^ ions, method developed by [50] was used. The protein was solvated with a simulation box of 7.7 × 11.8 × 8.2 nm^3^. To neutralize the system and to mimic experimental conditions, 21 Cl^−^ and 22 K^+^ were added by randomly replacing water molecules. In addition, one crystallographic Mg^2+^ ion was added to the catalytic site. To equilibrate water and ions, we restrained the position of the heavy atoms in the enzyme and conduct MD for 10 ns using constant pressure (NPT) simulation. The pressure was kept at 1 bar using Parrinello–Rahman scheme [42]. NPT simulation was followed by constant volume (NVT) simulation for another 10 ns with the position restrains. Unrestrained NVT simulations were later carried out for sampling the conformational states. In all simulations, Leapfrog integration scheme was used with a time step of 2 fs. The temperature was kept ~300 K using the Velocity-Scaling method implemented in Gromacs [51]. van der Waals interactions were calculated with 7–10 Å switching scheme. Electrostatic interactions were treated by Particle Mesh Ewald summation method with cubic interpolation order of 4 and grid spacing of 1.6 Å with a real-space cutoff of 12 Å. All bonds in the enzyme and water O–H bonds were constrained by LINCS algorithm. The dynamics of the enzyme were simulated for ~300 ns for the WT and R458A mutant. Gromacs suit of programs later used to perform trajectory analysis.

### Initial velocity and thermal inactivation studies

The HK2 reaction rate was measured spectroscopically using G6P dehydrogenase (G6PDH) couple reaction by monitoring NADH produced from the G6PDH reaction at 340 nm (*ε*_340_ =6220 M^−1^ cm^−1^) using a PerkinElmer Lambda 25 spectrophotometer equipped with a PTP-6 Peltier Temperature Programmer. The enzymatic reaction contained 20 mM HEPES (pH 7.5), 20 mM MgCl_2_, 3 mM NAD^+^, and 0.1 U/µl G6PDH (Sigma- G2921) at different concentrations of glucose and ATP from 0.5- to 10-times of their *K*_m_ values. All the data were plotted in a double reciprocal form to determine the quality of the data then fitted using (eqn 1) for a sequential mechanism.

(1) $$v\;=\;\frac{V_{\max}\;AB}{K_{a}K_{b}\;+\;K_{b}A\;+\;K_{a}B\;+\;AB}$$

where *v* and *V*_max_ are initial and maximum velocities, respectively, *A* and *B* are substrate concentrations, and *K*_a_ and *K*_b_ are Michaelis constants for substrates *A* and *B*, respectively. Data were fitted using the Global fitting analysis in the kinetics module of SigmaPlot (Systat Software, Inc. San Jose, California, U.S.A., www.sigmaplot.com).

The *T*_Opt_ was determined from the enzymatic activity of the different HK2 variants over a temperature range from 15 to 60°C. Reaction mixtures contained 10 mM HEPES pH 7.5, 1 mM ATP, 0.3 mM glucose, 1 mM NAD^+^, 20 mM MgCl_2_ were incubated at the specific temperatures for 3 min prior the addition of 0.1 unit/µl G6PD and 150 nM HK2 enzymes. The HK2 activity was monitored for 3 min at 340 nm. Error bars were calculated from triplicates of each reaction.

Thermal inactivation kinetics of HK2 at 37°C in the presence or absence of 5 mM glucose or 5 mM ATP were analyzed using two-state mechanism (N↔U). Enzyme aliquots at different time intervals were assayed as described above for residual activity and were plotted as a function of HK2 incubation time. Thermal inactivation was analyzed considering first order activity decay, and a semi-log plot of % residual activity was plotted as a function of HK2 incubation time. The enzymatic activity decay was fitted to a first order reaction, (eqn 2) or to a signal phase exponential decay using Prism 6, GraphPad Software.

(2) $$\ln[\text{A}]\;=\;-kt\;+\;\ln{\left[\text{A}\right]}_{0}$$

where *A_o_* and *A* are initial and remaining enzyme activity at different time intervals, respectively. *k* is the rate constant for enzyme inactivation and *t* is the incubation time. The half-life (t_1/2_) is the time required to decrease the enzyme activity by half, was calculated as ln(2)/*k*.

### Thermodynamic analysis using DSC

Calorimetric thermal unfolding measurements were performed on a Nano-DSC (TA instruments) with HK2 concentration between 0.3 and 0.8 mg/ml in a 20 mM HEPES pH 7.5 and 20 mM MgCl_2_ buffer solution. All samples were heated at a scanning rate of 1°C/min from 10 to 80°C under 3 atm pressure in the presence or absence of 5 mM glucose or 5 mM ATP. Background scans were obtained using buffer (with or without substrates) and collected same as above. DSC thermograms were corrected by subtracting the corresponding buffer baseline and converted into plots of excess heat capacity (Cp) as a function of temperature. The Δ*H*_cal_ was estimated from the area under the thermal transition and fitted using Nano Analyzer™ Software package from TA instruments.

### CD spectra and urea unfolding analysis

CD spectra were collected from 190 to 260 nm at 120 nm/s scanning speed and 1 nm bandwidth on a Chirascan™ CD spectrometer (Applied Photophysics) at 25°C using 1 mm quartz cuvette. Enzyme concentration was 0.2 mg/ml in a 50 mM phosphate buffer pH 7.5 in the presence or absence of 5 mM glucose or 1 mM ATP. CD measurements in the presence of ATP was carried out using 0.5 mm quartz cuvette.

The urea unfolding experiments were carried out using a CS/SF attachment on the Chirascan instrument at 222 nm with a path length of 2 mm and dead time of 3 ms. A circulating water bath maintained the temperature at 25°C and 10:1 mixing ratio was used to mix HK2 to a final concentration of 0.1 mg/ml at different urea concentrations. A minimum of four shots for each experimental condition was averaged to improve the signal-to-noise ratio. Unfolding data were fitted to a single exponential growth using the Pro-Data software from Applied Photophysics to acquire the *k*_u_ constants at different urea concentrations.

### ITD and DSF measurements

The rate of thermal unfolding was determined from ITD measurements by incubating HK2 in 50 mM phosphate buffer pH 7.5 at 37°C in the presence or absence of 5 mM glucose or 1 mM ATP. CD scans were collected every minute from 210 to 230nm for 50 min as described above. The protein (0.3 mg/ml) stability at 37°C was monitored at 222 nm and normalized to fraction unfolded using (eqn 3).

(3) $$F_{Unf}\;=\;\frac{\theta \;-\;\theta_{N}}{\theta_{D}\;-\;\theta_{N}}$$

where *θ is* the ellipticity of protein at specific time, and *θ*_N_ and *θ*_D_ are the ellipticities of native and unfolded states, respectively. The ellipticity of the native state (*θ*_N_) was obtained prior to incubating the enzyme at 37°C, and the fully denatured state (*θ*_D_) was obtained at the end of the measurement and after incubating the protein at 80°C for 1 h. The fraction of unfolded protein (*F*_Unf_) was fitted to straight line, where the slope was equal to the rate of thermal unfolding (*k*).

DSF was used to determine the HK2 thermal *T*_m_ using SYPRO Orange protein dye to monitor the protein unfolding on a Stratagene Mx3005P QPCR system (Agilent Technologies, La Jolla, CA) at *λ*_ex_ =492 nm and at *λ*_em_ =555 nm. To each well of a 96-well thin-walled unskirted PCR microplate (Bio–Rad, catalog number : 223 94444), samples consisting of 5 µl buffer (20 mM HEPES, pH 7.5 and 20 mM MgCl_2_), with or without 5 mM glucose or 5 mM ATP, 0.3 mg/ml protein sample, and 3× SYPRO Orange dye in a total volume of 10 µl. Fluorescent reads were collected from 25 to 80°C at 1°C/min temperature ramp rate. The data were fitted to a Boltzmann sigmoidal function to calculate the *T*_m_ at the middle of the transition using the Excel add-on package XLfit (IDBS limited, Bridgewater, NJ, U.S.A.) as described [52]. Final graphs were created using the GraphPad Prism 6 software (GraphPad Software, Inc. La Jolla, CA, U.S.A.).

The aggregation propensity of the HK2 variants was assessed by DSF using ProteoStat Protein Aggregation Assay (Enzo Life Sciences, PA, U.S.A.). The fluorescence intensity was recorded similar to DSF analysis in the presence of 3 mM Enzo dye in 96-well plates at temperature ramp rate of 1°C/min. *T*_agg_ was calculated at half the thermal transition.

## Results

Only HK1 and HK2 of the four human hexokinase isozymes bind to the OMM via their MBP at the N-terminus [24]. However, the structural fold, thermodynamic, and kinetic stabilities of the mitochondrial and cytosolic conformations of HK2 are yet to be determined. When bound to the mitochondria, the MBP points into the mitochondria away from the HK2 enzyme. For *in vitro* biochemical characterization of the mitochondrial conformation, a variant lacking the MBP, the first 16 amino acids of the N-terminus (Δ16), will mimic the HK2 conformation on the mitochondria (Supplementary Table S1). On the other hand, the FL variant of HK2 will resemble the cytosolic conformation, where the MBP is not interacting with the mitochondria and can bind to the enzyme.

All HK2 variants were expressed in *Escherichia coli* and purified to >90% purity based on Coomassie staining SDS/PAGE analysis, where gel band densitometry of HK2 variants was calculated using ImageJ software (Supplementary Figure S1A) [25]. The structural integrity of the HK2 variants was verified using far-UV CD analysis of protein conformation with spectra similar to predominantly α-helical proteins with two ellipticity minima at 208 and 222 nm (Supplementary Figure S1B).

### Structural characterization of the human HK2

**Figure 1:**
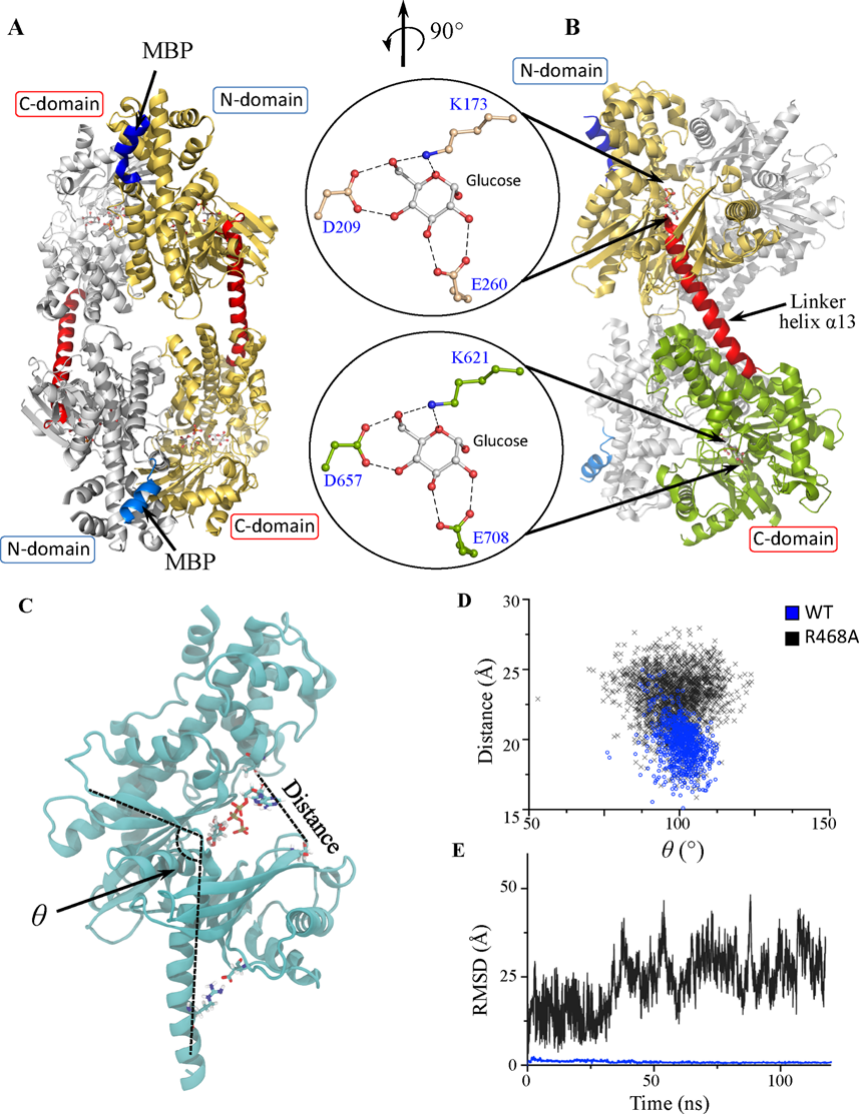
The crystal structure of human HK2 (**A**) Cartoon of the HK2 homodimer with monomers colored in white and gold. The MBP (blue) at beginning of the N-terminus and the linker helix-α_13_ (red) is between the N- and C-halves. (**B**) The monomer is divided into two structurally identical N- (gold) and C- (green) halves. The linker helix-α_13_ (red) is a long eight-turn helix that protrudes out of the active site of the N-half. **Insets**: the glucose-binding pockets in the N- and C-halves. (**C**) The open state of the N-terminus in complex with ATP and glucose obtained from molecular dynamics simulations (MD). The enzyme dynamics is projected on two order parameters where (*θ*) is the angle between helix-α_13_ and the large subdomain, and distance between T88 and T232 situated opposite sides of the active site. A salt bridge between R468-D202 dictates the position of helix-α_13_. (**D**) Correlation between the conformational parameters of WT (blue) and R468A (black). (**E**) The RMSD of glucose as a function of time for WT (blue) and R468A (black). This figure was prepared using PyMol (Schrodinger LLC) and VMD.

Multiple crystal screens were used for the crystallization of the different HK2 variants including FL, Δ16, and N- and C-domains for WT, D209A, and D657A mutants in the absence or presence of substrates, products, or their analogs. Only the Δ16 variant of the WT-HK2 crystallized in complex with glucose and G6P at 2.45 Å (Supplementary Table S2). The crystal structure of human HK2 is a homodimer with two structurally identical N- and C-halves of equal size linked by a long eight-turn linker helix-α_13_ (Figure 1 and Supplementary Figure S2). Similar to the overall fold of mammalian hexokinases, the N- and C-domains have an identical α/β fold that is conserved amongst the hexokinase family [16–23]. Each domain is distinctly folded into large and small subdomains that each contains a five stranded mixed β-sheet. The active site is formed by a cleft between the two subdomains, where their β-sheets enclose the active site and the helices α_5_ and α_13_ (Supplementary Figure S2A inset). The N-half of HK2 is linked to the C-half by helix-α_13_ that protrudes out of the active site of the N-domain. Helix-α_5_ that is perpendicular to the linker helix-α_13_, carries the catalytic residue D209 of the N-half. As we will show later, this network of interactions plays an important role in maintaining the catalytic activity of the N-domain of HK2.

Substrates binding to the apo-HK2 enzyme induced conformational transition to close the active site in preparation for catalysis, which was observed by a decrease in ellipticity after the addition of glucose in the CD spectra of HK2 (Supplementary Figure S1B). Atomically detailed molecular dynamics simulations (MD) were used to study the transition mechanism of HK2 during catalysis. The minimum energy path obtained using Steepest Descent Path (SDP, [26]) methodology suggests a collective motion of the two halves upon transition from open to closed states (Supplementary Movie S1, supplementary results). The opening and closing of the active site during catalysis is facilitated by movement of the small subdomains of the four halves of the homodimeric HK2 enzyme. During catalysis, the large subdomain does not move and its high conformational rigidity is achieved by the long ten-turn helix-α_11_ that spans the large subdomain (Supplementary Figures S2A,B).

The glucose-binding pockets in the N- and C-halves of HK2 are identical to other members of the hexokinase family, with the requirement of an aspartic residue for catalysis (Supplementary Figure S2C). Here, the double mutant, D209A and D657A, of the catalytic residues in the N- and C-halves of HK2, respectively, diminished the enzymatic activity of HK2. Since co-crystallization with ATP was not successful, the ATP-binding pocket of HK2 was modeled based on the crystal structure of HK4−ATP complex (Supplementary Figure S2C) [27,28]. The catalytic residue D209 or D657 of the N- and C-domains, respectively, forms H-bonding interactions with the 4-OH and 6-OH of glucose, and a lysine residue, K173 or K621, align the γ-phosphate of ATP with the 6-OH of glucose for its phosphorylation.

### The kinetic characterization of HK2 variants and the catalytic inactivation of the N-domain

The initial velocity patterns of the different HK2 variants were obtained in the forward direction by varying the concentrations of glucose at different fixed concentrations of ATP. The kinetic parameters obtained were similar to those reported previously (Supplementary Figure S1C−G and Table S3) [29,30], where the catalytic efficiency of the mitochondrial conformation Δ16-HK2 is higher than the FL variant, where the *V/K*_Glu_*E*_t_ and *V/K*_ATP_*E*_t_ of Δ16-HK2 was 0.6- and 2.0-fold higher than the FL-HK2, respectively.

The N- and C-domains of HK2 are split into large and small subdomains enclosing the active site and helices α_5_ and α_13_ (Supplementary Figure S2A,B). To kinetically characterize each domain separately, the N- (residues: 1–462) and C- (residues: 465–916) domains were expressed separately from the whole enzyme. Our initial design of the N-domain did not include the last four turns of the eight-turn linker helix-α_13_ at the end of the N-domain to avoid any structural misfolding of the domain. The last four turns of helix-α_13_ protrude outside the N-domain and are exposed to solution; therefore, it was not included in the initial construct design as not to disturb the structural fold of the N-domain.

The C-half was catalytically active, but to our surprise, the N-half with short four-turns of helix-α_13_ was completely inactive. The inactivation of the separate N-domain was not due to misfolded domain as CD spectral analysis (data not shown) confirmed its structural fold integrity. For this reason, a detailed kinetic study of different HK2 variants was necessary to validate our findings in comparison with what have been reported previously that the N-domain of HK2 is active [29,30]. The kinetic activity of N- and C-domains was also assessed in the FL and Δ16 enzyme variant by the introduction of a single mutation D209A or D657A one at a time, respectively (Supplementary Figure S1C−G and Table S3). The catalytic rate (*V/E*_t_) of the N-half (FL-D657A mutant) was 0.5-fold higher than the C-half (FL-D209A mutant) of HK2. However, the deletion of the MBP increased the *V/E*_t_ for the C-half (Δ16-D209A mutant) by two-fold in comparison with the N-half (Δ16-D657A mutant) (Supplementary Figure S1C−G and Table S3). These findings confirm that the N-half of HK2 is catalytically active and the inactivation of the separate N-domain observed here might be related to the initial design of the construct.

In the short variant of the N-domain (residues 1–462), part of the linker helix-α_13_ was not included in the construct design (Figure 1B and Supplementary Figure S2A). To investigate its role in the activity of the N-domain, different variants of the N-domain were constructed with increasing the length of helix-α_13_ to 6- (residues 1–469), and full eight-turns (residues 1–479). We found the activity of the N-domain to be dependent on the length of the helix, where longer helix-α_13_ promoted higher activity of the N-domain. The *V/E*_t_ of the separate N-domain was similar to the FL-D657A mutant and the C-domain was >three-fold slower than the FL-D209A mutants (Supplementary Figure S1E). These results confirm that the activity of the N-domain is dependent on the conformation of helix-α_13_ that is perpendicular to helix-α_5_ carrying the catalytic residue D209 of the N-domain (Supplementary Figure S2A inset). This network of interactions is important in determining the activity of the N-domain by the linker helix-α_13_.

All atom MD performed on the open state of HK2 confirmed the contribution of helix-α_13_ on the activity of the N-half, where it was stable with an average RMSD of 1.8 Å in 0.3 μs simulation time. One of the key interactions was the formation of a salt bridge between R468 on helix-α_13_ and D202 on the smaller subdomain of the N-half (Figure 1C). Local dynamics of the open state showed strong correlation between *θ*-angle and the active site width quantitated by the distance between T88 and T336 of the small and large subdomains, respectively (Figure 1C,D). While *θ*-angle was measured between helix-α_13_ and the first β-strand of the large subdomain, wide values of the *θ*-angle correlate with narrow groove width of the active site, and hence tighter H-bonding network between HK2 and the substrates, glucose, and ATP. Computationally, R468A mutant abolished the salt bridge with D202 and produced broader values for *θ*-angle with >10 Å wider active site and hence weaker H-bonding network with the substrates for weaker binding interactions. As a result, a rapid dissociation of glucose was observed in R468A mutant compared with the WT enzyme (Figure 1E). Similar results were obtained with truncated linker helix-α_13_ (residues: 1–462), where the salt bridge remotely modulates the substrates affinity (data not shown).

### Thermodynamic characterization of the HK2 variants and the mitochondrial-bound state

Multiple thermoanalytical techniques were utilized to characterize the cytosolic (FL) and mitochondrial (Δ16) conformations of HK2. The Δ16 variant lacking the MBP will mimic the enzyme’s conformation on the mitochondria. While, the FL variant will be similar to the cytosolic state, as the MBP is not interacting with the mitochondria and available to interact with the enzyme. The human HK2 enzyme has been shown previously to possess high catalytic activity when bound to the mitochondria [31]. Similarly, the Δ16 variant has been shown previously [29,30] and here to possess higher catalytic efficiency compared with the FL variants, which indicate that the Δ16 variant has a conformation similar to the enzyme on the mitochondria, and the FL variants will be similar to the cytosolic conformation.

**Figure 2:**
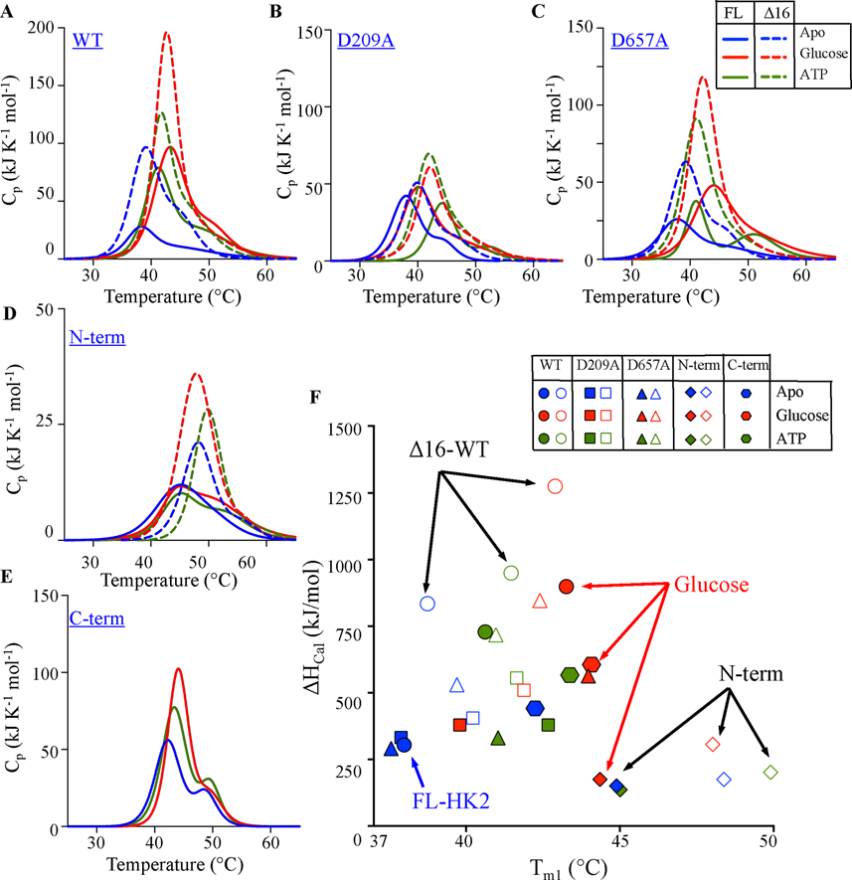
Thermodynamic analysis of HK2 (**A**–**E**) DSC thermograms of FL (solid bars) and Δ16 (dotted bars) variants of the WT, D209, D657A, and N-term of HK2 in addition to the C-term in the absence (blue) and presence of 5 mM glucose (red) or 5 mM ATP (green). DSC scans were corrected for buffer base line and data fitted to non-two-state transitions showing co-operative endothermic unfolding. (**F**) Correlation of the DSC parameters, Δ*H*_cal_ and *T*_m1_. The FL (solid symbol) and Δ16 (open symbol) variants of the WT (●), D209A (▪), and D657A (▲) mutants of HK2 are indicated in the background of N- (♦) and C-halves (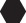). Data are mean ± S.D., *n*=3.

Differential scanning calorimetry (DSC) was used for detailed thermodynamic characterization of HK2 in the absence or presence of substrates (Figure 2). The overall shapes of the thermograms were similar for all HK2 variants with deconvolution patterns indicating a two-state transition with an early large and a late small melting transitions yielding two melting temperatures (*T*_m1_ and *T*_m2_). The calorimetric enthalpy (Δ*H*_cal_) was determined from the area under the thermographic peak, which corresponds to the total heat absorbed by the protein during the heat denaturation transition. Thermal unfolding of all variants was calorimetrically irreversible, and the addition of either substrate increased the *T*_m1_ and Δ*H*_cal_ by up to 6°C and 600 kJ/mol, respectively (Supplementary Figure S3A,B).

The mitochondrial conformation (Δ16) of HK2 possessed higher Δ*H*_cal_ value than the cytosolic conformation (FL) with minimal change on *T*_m1_ (Figure 2F). In the absence of substrates, deleting the MBP of HK2 increased Δ*H*_cal_ >500 kJ/mol with less than 1°C increase in *T*_m1_. In the presence of glucose, similar effect was observed with increase in Δ*H*_cal_ >350 kJ/mol and again negligible change in *T*_m1_. These findings are not commonly observed in proteins’ DSC thermograms, as an increase in ΔH_cal_ is usually accompanied by similar increase in *T*_m_.

The Δ*H*_cal_ value, calculated from the area under the thermographic peak, is related to the type of bonding interactions. An endothermic profile with a positive thermogram is a characteristic of H-bonding and polar interactions in the protein structure, but an exothermic pattern with negative thermogram is a result of breaking hydrophobic interactions in the protein structure as exposed to the aqueous environment [32,33]. The increase in the Δ*H*_cal_ value upon the deletion of the MBP (Δ16) suggests that the hydrophobicity of HK2 decreased. This suggests that upon binding to the mitochondria, conformational changes alter the intermolecular interactions of HK2 to be more optimum for a cytosolic environment. The low total protein yield from *E. coli* expression for all the FL variants was at least one-fold lower than the Δ16 variants of HK2 (Supplementary Figure S1H). The increased aggregation on the gel filtration profiles (data not shown) explain the low protein expression yield for the FL variants and support its low Δ*H*_cal_ values as a result of increased hydrophobicity. Furthermore, the high protein yield of Δ16-HK2 and its low hydrophobicity indicate conformational changes upon translocation to the mitochondria, where the enzyme possesses more hydrophobic character. The thermodynamically most stable enzyme was the Δ16 variant of the WT in the presence of glucose, which indicates the translocation and high occupancy of HK2 on the mitochondria can be driven by higher conformational stability.

To determine the thermodynamic contribution of each domain on the stability of the entire enzyme, glucose binding was assessed on each domain after mutating its catalytic residue one at a time in the whole enzyme. For example, the thermodynamic contribution of the C-domain was characterized from the FL and Δ16 variants of the D657A mutant, where the glucose-binding pocket of the C-domain was altered. The overall shapes of the D657A thermograms of the FL and Δ16 variants in the absence or presence of substrates were similar to the WT enzyme with similar *T*_m1_ but lower Δ*H*_cal_ values (Figure 2A,C). Similar to the WT enzyme, the addition of glucose increased *T*_m1_ and Δ*H*_cal_ of D657A variants from 2.7 to 6.4°C and from 270 to 590 kJ/mol, respectively (Supplementary Figure S3A,B). On the other hand, mutating the catalytic residue D209A of the N-domain produced thermograms that are different from the WT enzyme (Figure 2B). The addition of glucose moderately increased the thermodynamic stability of the D209A mutant with a subtle increase in *T*_m1_ and Δ*H*_cal_ of less than 2°C and 104 kJ/mol, respectively.

The addition of glucose increased the thermodynamic stability for all HK2 variants except the D209A mutant as well as the N-domain (Figure 2B,D). The *T*_m1_ of the N-domain did not change in the presence of glucose for the FL and Δ16 variants of the N-domain. However, the deletion of the MBP of the N-domain increased its *T*_m1_ from 45 to 48.5°C, respectively (Supplementary Figure S3A,B). These findings highlights the role of the N-half in controlling the stability of the whole enzyme, where mutating the catalytic residue of the N-domain was sufficient to eliminate the glucose stabilization effect on the entire enzyme in addition to the higher thermodynamic stability of the N-domain after truncating the MBP.

In the addition to DSC analysis, differential scanning fluorimetry (DSF) was implemented to examine the global thermal unfolding of HK2 variants in the presence of SYPRO Orange reporter dye (Supplementary Figure S3C). Similar to DSC analysis, the addition of glucose increased the melting temperature (*T*_m_) of FL and Δ16-HK2 from 34 to 42°C for WT and D657A variants, but it did not enhance the stability of the D209A mutant (Supplementary Figure S3C). Interestingly, the deletion of the MBP substantially increased the stability of the N-domain in the presence or absence of glucose. A direct correlation was observed between *T*_m_ and *T*_m1_ obtained from DSF and DSC analyses, respectively (Supplementary Figure S3E). However, less correlation was observed between *T*_m_ and Δ*H*_cal_ values from DSF and DSC analyses, respectively (Supplementary Figure S3F). To confirm whether the DSF data were direct measure of protein unfolding and not aggregation, an Enzo ProteoStat detection dye was used to determine the aggregation temperature (*T*_agg_) of HK2 variants at half thermal transition (Supplementary Figure S3D). The thermal unfolding preceded domain aggregation since *T*_agg_ was >5°C higher than *T*_m_ for all HK2 variants except the N-domain that was more aggregate prone.

### The kinetic stability, optimum temperature of catalysis, and half-life of HK2 variants

**Figure 3:**
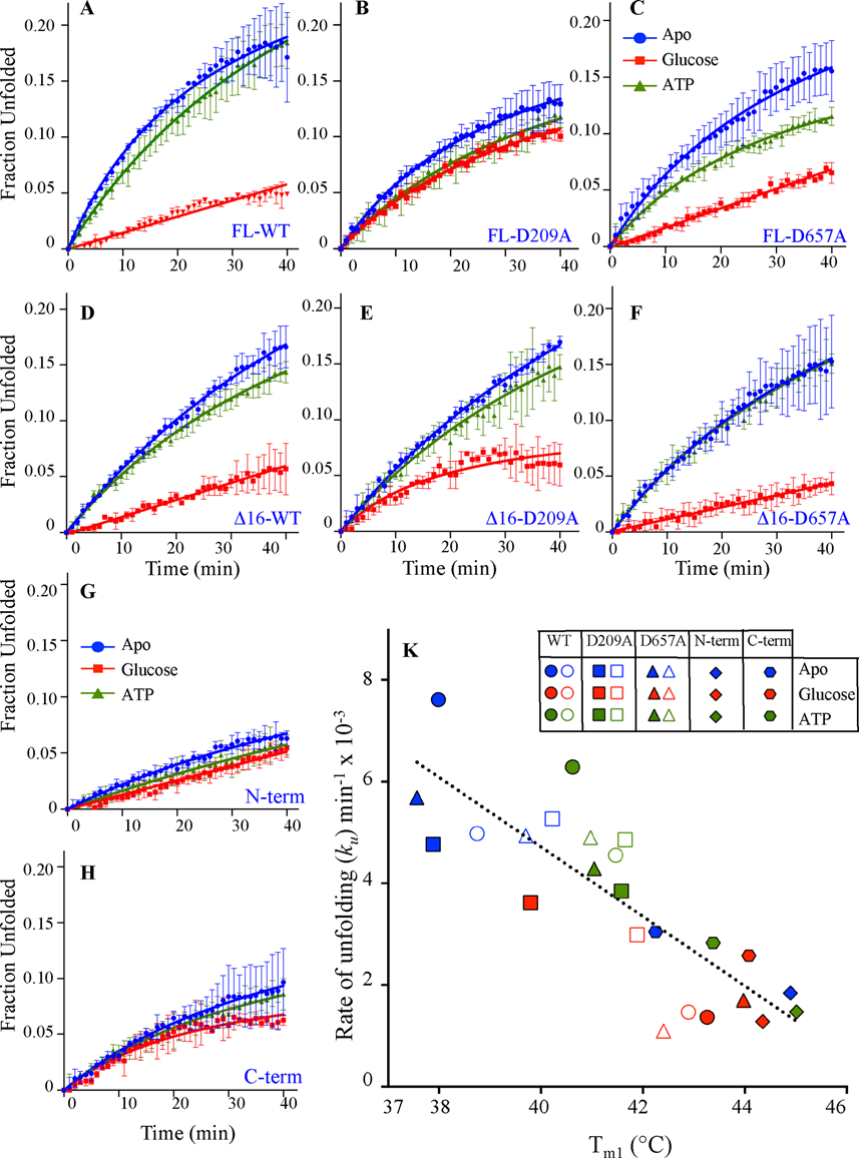
Thermal kinetic stability of HK2 at 37°C from isothermal denaturation analysis (**A**–**H**) Time dependence of CD scans on the thermal kinetic stability of HK2 variants in the absence and presence of 5 mM glucose or 1 mM ATP at 37°C. Ellipticity readings at 222 nM were normalized to fraction unfolded protein. Thermal unfolding rates (*k*_u_) were calculated from the slope of the lines. (**K**) Correlation between *k*_u_ measured here and *T*_m1_ from the DSC analysis. Data are mean ± S.D., *n*=3. Symbols and colors are as in Figure 2.

The thermal kinetic stability of HK2 variants was determined using isothermal denaturation (ITD) by monitoring the unfolding of HK2 at 37°C in the absence or presence of substrates (Figure 3). The unfolding rate (*k*_u_) was calculated from monitoring the enzyme denaturation at 222 nm in a CD spectrophotometer. The unfolded fraction was calculated from comparison of the fully unfolded and folded states. In the absence or presence of ATP, the Δ16 variant of the WT enzyme was more stable than the FL-HK2. The deletion of the MBP decreased the *k*_u_ of the WT enzyme and in the presence of glucose, both had similar kinetic stabilities (Supplementary Figure S4A).

In the absence of substrates, the separate N- and C-domains were the most stable with low *k*_u_ values (Supplementary Figure S4A). As observed with the DSC and DSF analyses, the addition of glucose increased the kinetic stabilities of the WT and D657A variants from 2.0- to 4.6-fold as a result of decrease in their *k*_u_. However, the addition of glucose increased the kinetic stability of the D209A mutant of Δ16- and FL-HK2 by less than one-fold. This again underlines the role of the N-domain in stabilizing the whole enzyme, where a direct correlation was observed between *k*_u_ obtained here and *T*_m1_ from DSC analysis (Figure 3K). The kinetic and thermodynamic stabilities of all HK2 variants increased upon the addition of glucose, except the D209A mutant.

**Figure 4:**
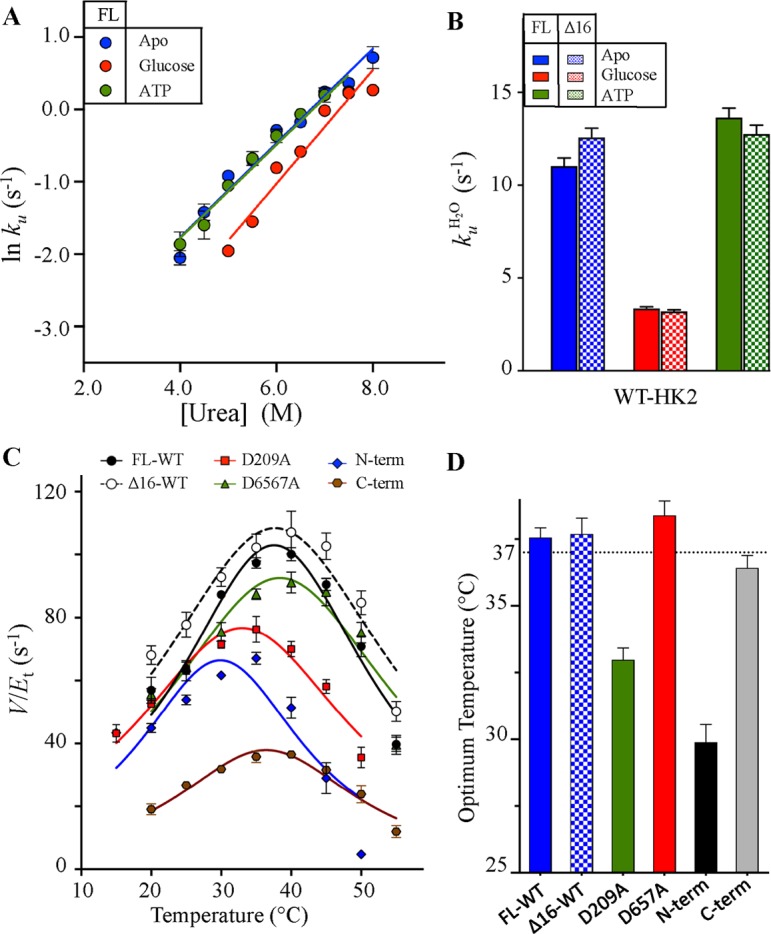
Chemical kinetic stability and *T*_Opt_ of HK2 (**A**) The rate of unfolding (*k*_u_) of the FL- and Δ16-HK2 variants of the WT were determined as a function of urea concentration. *k*_u_ was monitored at 222 nm using stopped-flow CD spectroscopy at 25°C in the absence (blue) and presence of 5 mM glucose (red) or 1 mM ATP (green). (**B**) The *k*_u_ in water (*k_u_*_H2O_) of the FL- (solid bars) and Δ16- (checkered bars) HK2 variants was calculated after extrapolating the rate to zero urea concentration for experiments in (B). (**C**) The temperature dependence of the specific enzymatic activity of HK2 variants. (**D**) The *T*_Opt_ calculated at the middle of the peak for variants in (C). Data are mean ± S.D., *n*=3. Abbreviation: *T*_Opt_, optimum temperature of catalysis .

The chemical kinetic stability of the WT HK2 was also measured as a function of urea concentration using stopped-flow CD spectroscopy (Figure 4A). The urea unfolding kinetics exhibited a single exponential pattern with the *k*_u_ in water (*k_u_*_H2O_) determined from extrapolating the rate to zero urea concentration. Similar to the ITD results, the chemical kinetic stabilities of the WT variants increased upon the addition of glucose with more than three-fold decrease in *k_u_*_ H2O_ (Figure 4B). The deletion of the MBP did not affect the kinetic stability of the Δ16 variants in comparison with the FL-HK2.

The thermal stability of HK2 can also be assessed as a function of its catalytic rate (*V/E*_t_). The optimum temperature of catalysis (*T*_Opt_) was determined at the middle of the bell-shaped temperature profile with temperature correlated to the highest *V/E*_t_ rate for each variant tested. The temperature dependence on *V/E*_t_ for all HK2 variants was similar with *T*_Opt_ at physiological temperature of 37°C except for the N-domain and D209A mutant with *T*_Opt_ values of 30 and 33°C, respectively (Figure 4C,D). Since HK2 activity was measured in the presence of glucose, a direct correlation was observed between *T*_Opt_ and *T*_m1_ values determined from DSC analysis of HK2 in the presence of glucose (Supplementary Figure S4B). The *T*_Opt_ value of the D657A mutant was similar to the WT enzyme, where mutating the catalytic residue of the C-half did not alter the *T*_Opt_ of D657A mutant. However, the D209A mutant of the N-domain lowered the *T*_Opt_ consistent with its low thermodynamic stability (*T*_m1_) in the presence of glucose, which supports the regulatory role of the N-half in the function and stability of the whole HK2 enzyme.

As for the separate domains that possessed high thermodynamic stabilities based on multiple thermoanalytical analyses, the *T*_Opt_ of the C-domain was similar to the WT enzyme (Figure 4C,D). However, the N-domain possessed the lowest *T*_Opt_ value even though its thermodynamic stability was similar to the C-domain and the FL-enzyme (Supplementary Figure S4B). The high stability of the N-domain confirms that the loss of activity at lower *T*_Opt_ is not due to structural integrity of the enzyme rather due to loss of conformation of the partially exposed helix-α_13_ of the N-domain. From initial velocity studies, we showed the importance of helix-α_13_ in maintaining the catalytic activity of the N-domain. More than half of helix-α_13_ is exposed to solvent and protrudes out of the active site of the N-domain. The low *T*_Opt_ value of the N-domain is likely due to destabilization of the linker helix-α_13_ at a lower temperature that resulted in an early loss of the catalytic activity.

**Figure 5:**
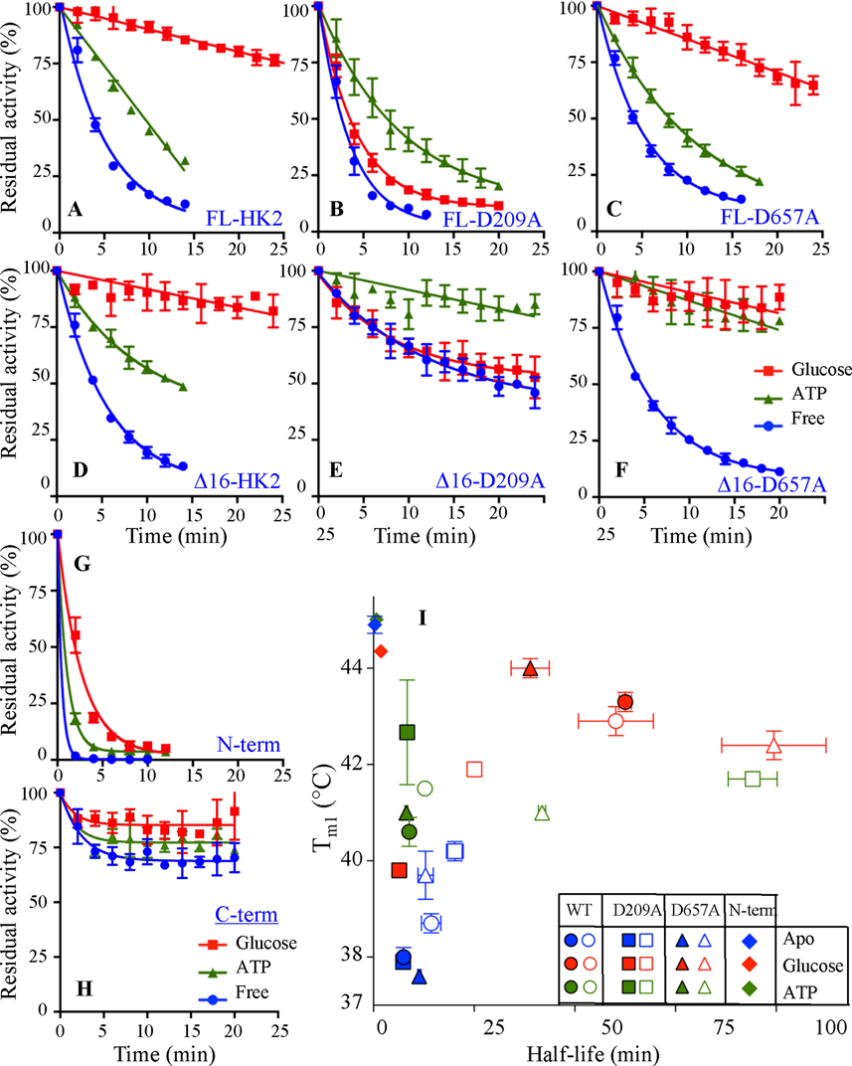
Half-life (t_1/2_) calculation from thermal inactivation kinetics of HK2 (**A–H**) The residual enzymatic activity of HK2 variants at different time intervals after incubating the enzyme in the absence or presence of substrates at 37°C. (**I**) Correlation between t_1/2_ and *T*_m1_ from DSC analysis of the different HK2 variants. Data are mean ± S.D., *n* 3. Symbols and colors are as in Figure 2.

Furthermore, to establish the relation between the stability of HK2 and its catalytic function, the time dependence on the residual catalytic activity of HK2 was determined at 37°C in the absence or presence of substrates (Figure 5). The enzyme was incubated at physiological temperature in the presence or absence of substrate and its catalytic rate (*V/E*_t_) was measured at different time intervals. The kinetic half-life (t_1/2_) of the HK2 variants was determined from the time-dependent decrease for half the catalytic activity. A fast catalytic inactivation was observed for all HK2 variants in the absence of substrates with a t_1/2_ of less than 10 min (Supplementary Figure S4C). The addition of glucose increased the t_1/2_ by 10- to 24-fold for the FL and Δ16 variants of WT and D657A mutant, but it did not increase the t_1/2_ for the D209A mutant. Again, mutating the catalytic residue of the N-half compromised the glucose stabilization effect on HK2. This was not the case for the D657A mutant, where an intact glucose binding pocket in the N-half was sufficient to stabilize and maintain the catalytic activity for >40 min compared with <6 min for the D209A mutant.

Correlating the t_1/2_ with *T*_m1_ obtained from DSC analysis revealed enhanced catalytic stability only for HK2 variants with *T*_m1_ values above the physiological temperature (Figure 5I). The C-term lost only 30% of its catalytic activity after 40-min incubation at 37°C (Figure 5H). Even though the separate domains were thermodynamically most stable variants, the N-term was completely inactive in less than 5 min (Figure 5G). Similar to its low *T*_Opt_, the N-domain short t_1/2_ is due to early destabilization of the partially solvent exposed linker helix-α_13_ at the end of the N-domain that is essential for its catalytic activity.

## Discussion

The uncontrolled and rapid proliferation of cancer cells is initiated after a switch in glucose metabolism from mitochondrial oxidative phosphorylation to aerobic glycolysis known as the ‘Warburg effect’ [1,2]. Glucose metabolism is elevated during the transformation of normal to malignant cells, an important hallmark of tumors that up-regulate HK2 expression [24]. The dominant presence of HK2 on the mitochondria and the regulatory role of its N-domain in the inhibition of apoptosis, highlight the molecular and structural importance for the characterization of HK2 roles in the initiation and maintenance of cancer [14,15]. In this study, biochemical and structural characterization of the cytosolic (FL) and mitochondrial (Δ16) conformation of HK2, proposed a mechanism for the translocation of HK2 to the mitochondria. In addition, the conformation of helix-α_13_ that links the N- with C-half of HK2 was found to be important for maintaining the catalytic activity of the N-domain as observed experimentally and from computational modeling. Further characterization of the single mutants, D209A or D657A, in HK2 reveals the regulatory role of the N- but not the C-domain in the thermodynamic and kinetic stabilities as well as the half-life of the whole enzyme.

### Enhanced conformational stability and catalytic activity of the mitochondrial conformation of HK2

The mitochondria, the site for energy metabolism and regulation of cell death, plays an important role in tumorigenesis and metastasis. Of all the enzymes in the glycolytic pathway, and of the four human hexokinase isozymes, only HK1 and HK2 bind to the mitochondria for enhanced catalytic activity and relief of feedback inhibition by its product, G6P [29,30]. In tumor cells, HK2 is predominantly localized on the OMM, where its translocation is facilitated by an increase in the cellular glucose concentration [11–13].

The FL and Δ16 variants that mimic the cytoplasmic and mitochondrial conformations of HK2, respectively, are used to characterize the molecular machinery and forces required for HK2 translocation to the mitochondria. Since HK2 attaches to the OMM via its MBP, the conformation of Δ16 variant lacking the MBP at the beginning of its N-terminus will mimic the mitochondrial conformation of HK2. Enhanced conformational stabilities of Δ16-HK2 variants in comparison with the FL-HK2 with >two-fold increase in Δ*H*_cal_ was observed from DSC analysis with limited change in *T*_m1_ values. The increase in Δ*H*_cal_ values after the deletion of the MBP is due to induced conformational changes that decrease the hydrophobicity of the FL- compared with the Δ16 variants of HK2. In general, proteins with polar character yield positive endothermic peaks on the DSC thermograms, but negative exothermic peaks are observed for hydrophobic proteins [32,33]. These findings were also confirmed by the higher protein yield after *E. coli* expression for all Δ16 variants with less aggregated samples in comparison with the FL-HK2. In addition and in the absence or presence of ATP, the rate of thermal unfolding measured from ITD analysis was lower for the Δ16-HK2 variants compared with FL enzyme. As a result, the mitochondrial conformation of HK2 is kinetically and thermodynamically more stable, which supports the notion that the high occupancy of HK2 on the mitochondria and its translocation from the cytoplasm is due to higher stability.

The addition of glucose increased the thermodynamic stability of all HK2 variants with the highest thermodynamic and conformational stabilities observed for the Δ16 variant of the WT enzyme. Similarly, glucose decreased the chemical and thermal *k*_u_ of HK2 making it kinetically a more stable enzyme. These results support previous reports of high occupancy of HK2 on the mitochondria [11,12], where the translocation is facilitated by a more stable conformation on the mitochondria.

### The catalytic activity of the N-domain of HK2 is regulated by the linker helix-α_13_

The human hexokinase 1, 2, and 3 contain two domains equal in size and structurally similar, where each domain contains a conserved glucose-binding pocket with the requirement of an aspartic residue for catalysis [18,28]. Regardless of their high structural similarity and active site identity, the N-domains of HK1 and HK3 are catalytically inactive with previous reports attributing the inactivation of the N-domain to regulatory roles they play in the overall function of the enzymes [16,34]. Currently, there are no mechanistic evaluation for the molecular inactivation of the N-domain of HK1 and HK3, and from a structural and biochemical point of view, the N-domains of the human hexokinase isozymes should all be catalytically active.

Here and for the first time, we identified an inactive variant of the N-domain of HK2 in an attempt to describe the mechanism by which other hexokinase isozymes lost activity in their N-domains. We found the size of the linker helix-α_13_ to be essential in maintaining the catalytic activity of the N-half of HK2. In hexokinase 1 and 2, helix-α_13_ is a long eight-turn helix that extends out of the active site of the N-half to link it to the C-half (Figure 1B and Supplementary Figure S2A) [16,17]. To express the N-half separately from the enzyme, multiple variants have been constructed with different sizes of the linker helix-α_13_ including four-, six-, and eight-helical turns. To our surprise, the N-half with short four-helical turns of helix-α_13_ was completely inactive. However, the N-half of HK2 is catalytically active based on initial velocity studies of the D657A mutant that contain inactive C-domain. Furthermore, the catalytic activity of the N-domain was restored upon increasing the size of helix-α_13_ with the highest activity recorded for N-domain with the full eight-turn helix. These results show that the activity of the N-domain of HK2 is dependent on the conformation of its linker helix-α_13_.

MD of the HK2 reaction mechanism revealed distinct conformational changes along the linker helix-α_13_ (Supplementary Movie S1), where opening the N-half was possible with the bending of helix-α_13_. Even though the structural folds of their domains are identical, overlay of the entire homodimers of HK1 (PDB code: 1HKB) [18] with HK2 in complex with glucose and G6P were not possible. However, alignment was possible only through one domain but not both, where alignment on the N-domains reveals a 16° bent on helix-α_13_ of HK1 compared with HK2 (Supplementary Figure S2D). From the HK2 simulation (Supplementary Movie S1), the linker helix-α_13_ underwent conformational changes for the opening and closing of the active site of the N-domain. The bent conformation of helix-α_13_ in HK1 and the stiffness of its backbone may hinder the opening of its active site in the N-domain.

### The N-domain but not the C-domain regulates the stability and activity of the entire HK2 enzyme

The catalytic role of HK2 in glucose intracellular retention and metabolism is critical for cancer cells survival and proliferation, where its binding to the OMM provides a preferential access to mitochondrial ATP needed for catalysis and evades feedback inhibition by its products, G6P [14,35]. The mitochondria binding of HK2 is also important to suppress apoptosis and cancer cell death [14,15]. It has been reported previously and confirmed here that an increase in the catalytic efficiency of HK2 was accomplished upon translocation to the mitochondria, where the catalytic efficiency of Δ16 was higher than the FL variants [29,30]. However, these findings do not explain the high occupancy of HK2 on the mitochondria.

To investigate the regulatory role of the N-half on the stability of HK2, an alanine mutation was introduced at the catalytic residue of the N- (D209) or C- (D657) domains to inactivate one domain at a time. The FL variants of the WT, D209A, and D657A of HK2 had similar kinetic and thermodynamic stabilities in the absence of substrates (Figures 2F and 5I). However, the deletion of the MBP (Δ16-HK2) or the addition of glucose increased the stability of all HK2 variants, except the D209A mutant of the N-domain. As a result, an intact glucose-binding pocket in the N-domain is required for HK2 to adopt a stable conformation in the presence of glucose. Since the mitochondrial interaction is initiated by the N-domain of HK2, its role in maintaining the stability of HK2 enzyme explain mechanistically how the enzyme shifts its conformational stability upon binding to the mitochondria.

The evasion of cell death is one of the major causes of the failure of anticancer therapy. HK2 promotes tumor survival and resilience of cell death, where its mitochondrial dissociation was found to trigger apoptosis in cancer cells [14]. Therefore, the development of therapeutics that target HK2 specifically and facilitate its mitochondrial dissociation or inhibition of its catalytic activity will make an effective anticancer therapy. Therapeutics with high specificity for HK2 and low interactions with other human hexokinase isozymes will reduce the side effects of the new anticancer treatment. Detailed structural and biochemical analyses of the human hexokinase isozymes are necessary for the identification of unique characteristics of HK2 that can be used in the development of new anticancer drugs.

One of the major differences between HK2 and other human hexokinase isozymes is the loss of catalytic activity at the N-domain in HK1 and HK3. Here, we identified the role of helix-α_13_ that links the N- and C-halves of HK2 in maintaining the catalytic activity of the N-domain. Reducing the size of helix-α_13_ decreased or completely abolished the catalytic activity of the N-domain of HK2. These findings highlight the molecular mechanism for the metabolic adaptation of tumor cells in reprogramming their metabolism to meet the high demands of cancer cells.

## Supporting information

**Supplementary Figure 1:** Structural and catalytic characterization of human HK 2 variants. **(A)** SDS-PAGE analysis of the FL- and Δ16-HK2 variants of the WT, D209, and D657A as well as the separate N- and C-domains. Coomassie-stain was used to visualize the protein bands and ImagJ [18] was used to quantity purity >90%. **(B)** Far-UV CD spectra of the FL (solid line) and Δ16 (dashed line) variants of the WT-HK2 in the absence (blue) and presence of glucose (red). The CD spectra overlapped with minima at 208 and 222 nm. **(C-D)** IIIIIIIII **(E-G)** Kinetic parameters of the FL (solid bars) and Δ16 (checkered bars) variants of HK2 including different sizes of the N-domain including residues 1.469 (pink) and 1.479 (red). The rate was determined in the direction of formation of G6P. **(H)** Total protein yield of the FL- (solid bars) and Δ16- (checkered bars) HK2 variants per one liter of *E. coli* culture after completing all required purification steps.

**supplementary Figure 2:** The Crystal Structure of Human HK2. **(A-B)** The N- and C-halves of HK2 with large (white) and small (gold) subdomains. The MBP of the N-half is dark blue. Helix α_13_ (red) protrudes out of the active site at the end of the N- and C-halves. Two 5 stranded β-sheets (blue) encloses the active site in addition to helices α_5_ (green) and α_13_. **Inset**: Helix α_5_ that carries the catalytic residue D209 or D657 of the N- and C-halves, respectively, is perpendicular to helix α_13_. **(C)** The glucose (white) and ATP (yellow) binding pockets in HK2 with the later modeled based on the crystal structure of HK4.ATP complex. **(D)** Overlay of the monomers of HK1 (aquamarine) and HK2 (yellow) in complex with glucose and G6P. Alignment was only possible on N-half but not the FL enzyme. Even though the structural folds of HK1 and HK2 domains are identical, a 16° bent on the linker helix of HK1 prevented its alignment to HK2. This figure was prepared using PyMol (Schrodinger LLC).

**supplementary Figure 3:** Thermodynamic parameters of HK2 variants. **(A-B)** DSC parameters of FL (solid bars) and Δ16 (dotted bars) variants of the WT, D209, D657A, and N- and C-domains of HK2. T_m1_ was calculated from the temperature at the middle of the first transition, and ΔH_cal_ was determined from the area under the thermographic peaks of the DSC thermograms in figure 2. **(C-D)** DSF analysis of HK2 variants for the determination of T_m_ and T_agg_ in the presence of SYPRO Orange or Enzo ProteoStat reporter dyes, respectively. To confirm thermal unfolding preceded domain aggregation, ΔT (T_agg_ − T_m_) was >5°C for all variants except the Δ16 variant of N-term due to its high T_m_ value. **(E-F)** Correlation of the T_m_ from DSF analysis against DSC parameters, ΔH_cal_ and T_m1_, respectively. The FL (solid symbol) and Δ16 (open symbol) variants of the WT (●), D209A (▪), and D657A (▲) mutants of HK2 are indicated in the background of N- (♦) and C-halves (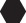). Data are mean ± SD, *n*=3.

**supplementary Figure 4:** Thermal Kinetic Stability, T_Opt_, and Half-Life of HK2 variants. **(A)** The thermal rates of unfolding (*k*_u_) of the HK2 variants FL (solid bars) and Δ16 (checkered bars). The *k*_u_ was measured after incubating HK2 at 37 °C in absence (blue) and presence of 5 mM Glucose (red) or 1 mM ATP (green) from the CD ellipticity readings at 222 nM. **(B)** Correlation between the optimum temperate of catalysis (T_Opt_) and T_m1_ from the DSC analysis in the presence of 5 mM glucose. A direct correlation is observed for all HK2 variants with the lowest values recorded for D209A mutant except for the N-term did with very low T_Opt_ value in comparison to its high thermal stability, T_m1_. **(C)** Half-life of HK2 variants measured by thermal inactivation kinetics. Symbols and colors are as in B. Data are mean ± SD, n=3.

**Supplementary Table 1:** HK2 variants.

**Supplementary Table 2:** Crystallographic data and refinement statistics, related to Figure 1, and Supplementary Figure 2. Numbers in parentheses represent the highest resolution bin.

**Supplementary Table 3:** Kinetic parameters for HK2 variants were determined in the direction of formation of G6P at 25 °C and pH 7.5, related to Supplementary Figure 1.

## Abbreviations

BTP: Bis-Tris propane
DSC: differential scanning calorimetry
DSF: differential scanning fluorimetry
FL: full-length
G6P: glucose-6-phosphate
G6PDH: G6P dehydrogenase
HK2: hexokinase 2
ITD: isothermal denaturation
*k*_u_: unfolding rate
MBP: mitochondrial-binding peptide
MD: molecular dynamics simulation
OMM: outer mitochondrial membrane
SDP: steepest descent path
SMOG: structure-based model for biomolecule
*T*_agg_: aggregation temperature
*T*_m_: melting temperature
TMD: targetted molecular dynamics
*T*_Opt_: optimum temperature of catalysis
WT: wild-type
Δ*H*_cal_: calorimetric enthalpy
