# Supplementary material for: The catalytic inactivation of the N-half of human hexokinase 2 and structural and biochemical characterization of its mitochondrial conformation
Source: Biosci Rep. 2018 Feb 21;38(1):BSR20171666. doi: 10.1042/BSR20171666 (PMC5803496; doi:10.1042/BSR20171666)
Supplement: Supplementary file 1 [file bsr20171666_Supp1.pdf]

Supplementary Material.

The Catalytic Inactivation of the N-half of Human Hexokinase 2 and Structural and Biochemical Characterization of its Mitochondrial Interactions.

Mir Hussain Nawaz[1,†], Juliana C. Ferreira[1], Lyudmila Nedyalkova[2,3], Haizhong Zhu[2], César Carrasco-López[1], Serdal Kirmizialtin[1], Wael M. Rabeh[1,†,*]

[1]Science Division, New York University Abu Dhabi, PO Box 129188, Abu Dhabi, United Arab Emirates.
[2]Structural Genomics Consortium, University of Toronto, Toronto, Ontario M5G 1L7, Canada.
[3]Present address: The Donnelly Centre, University of Toronto, Toronto, ON M5S 3E1, Canada.
[*]Corresponding author: wael.rabeh@nyu.edu

[†]These authors contributed equally to this work.

Supplementary Results

Minimum Energy Pathway of conformational transition from open to closed state.

Atomically detailed simulations were used to study the mechanism of the open to closed conformational transition for the FL-HK2 enzyme. By fixing the two end states at open and closed states, the minimum energy path determined by connecting the two states using steepest descent path (SDP) methodology [1]. The enzyme undergoes a collective motion during the conformational transition. The opening and closing of the four active sites are triggered by the movement of the small subdomains of the homodimeric HK2 that point to the outside of the enzyme.

To study the role of helix-α_{13} in catalysis and its key interactions with the enzyme surface, all atom molecular dynamics simulations (MD) were performed in explicit water and ions. The open state stayed stable during 0.3 μs simulation time with an average RMSD value of 1.8 Å. Local dynamics in the open state can be monitored with the fluctuation of the angle and the groove width of the active site (Figure 1D). The change in the groove width was estimated from the distance between T88 and T336.

A salt bridge was detected between R468 on the linker helix-α_{13} and D202 on the β-sheet of the small subdomain of the N-domain (Figure 1C). To decipher the effect of salt bridge on helix-α_{13} angle and overall to conformation of HK2 enzyme, MD simulations were conducted on the mutant R468A to determined its effect on the enzyme conformation and the binding stabilities of the substrates. Abolishing of the salt bridge between R468A and D202 leads to a wide distribution of θ angle in the R468A mutant in comparison to the WT enzyme (Figure 1C and 1D). Lower values of the θ angle lead to widening of the active site and hence weakening of the H-bonding network with the substrates, resulting in a rapid dissociation of the glucose from the active site of R468A mutant (Figure 1E). The salt bridge is proposed to remotely modulate glucose binding by widening the grove width of the active site.

Supplementary Methods

Modeling the open state of HK2.

The crystal structure of the HK2 enzyme was determined in the closed state. The apo-enzyme could not be crystalized from all the different variants constructed here and could not acquire the open conformation of HK2. Computational homology modeling was used to generate the open state of HK2. The structure of yeast hexokinase yeast [2] was used as a template in MODELLER [3] then targeted molecular dynamics (TMD) simulation protocol [4] implemented in Gromacs 4.05 [5] was used with the structure-based model for biomolecules (SMOG) potential [6]. SMOG potential allowed rapid sampling of the conformational space while keeping the secondary structure stoichiometry intact during the closed to open transition. The approach has been utilized heavily to study biomolecular processes including the mechanism of conformational transitions of large biomolecular complexes [7]. A TMD simulation of 250000 steps in reduced temperature was used to obtain the open conformation of HK2. The temperature was kept constant using velocity verlet scheme [8]. Nonbonded interactions were treated with 15Å cutoff. Stochastic dynamics integrator with a time step of 0.001 and a friction coefficient of 0.1 was used to integrate the equations of motion. The trajectory created with TMD later used to study the minimum energy pathway of the transition.

Modeling the transition pathway.

The 96 configurations equally spaced from initial TMD trajectory and minimized each using a classical Molecular Mechanics force field. The pathway from closed to open was refined using SDP methodology [1] implemented in MOIL [9]. In this approach given the two ends configurations R_C and R_O, a discrete set of coordinates between the two end states R_i where index $i = 2,3..,N - 1$ with the optimized the function $S[R(l)] = \int_{R_C}^{R_O} dl \sqrt{\nabla U^t \nabla U}$.

Here $R(l)$ is the coordinate vector as a function of arc-length l. $\nabla U(R(l))$ is the gradient of the potential energy. GBSA implicit solvent model [10] and OPLSAA force field was used to account for the interactions of glucose with the protein [11]. ATP parameters were adopted from previous work [12] with minimization protocol similar to work done previously [12]. Minimum energy path of closed to open state is shown in *Movie 1*. The trajectory was smoothed by averaging two consecutive frames for visualization purposes.

Molecular Dynamics Simulation Protocol.

Explicit water All Atom Molecular Dynamics Simulations were carried out in Gromacs 4.05 suit of programs [5]. OPLSAA [13] forcefield is used for protein and glucose. ATP parameters were adopted from [12]. Water is modeled using SPCE [14]. Smith and Dang parameters [15] were used for K^+ and Cl^-, and Mg^{2+} coordinates were acquired from [16]. The protein was solvated with a simulation box of 7.7 x 11.8 x 8.2 nm^3. To neutralize the system and to mimic experimental conditions, 21 Cl^- and 22 K^+ ions were added by randomly replacing water molecules. To equilibrate water and ions, the positions of the heavy atoms on the enzyme were restrained then run molecular dynamics simulation for 10 ns using constant pressure (NPT) simulation. The pressure was kept at 1 bar using Parrinello–Rahman scheme [17]. NPT simulation was followed by constant volume (NVT) simulation for another 10 ns with the position restrains. Unrestrained NVT simulations were later carried out for sampling the conformational states. In all simulations, Leap Frog integration scheme was used with a time step of 2 fs. The temperature was set to 300K using the Velocity-Scaling method implemented in Gromacs [8]. Van der Walls interactions were calculated with 7-10 Å switching scheme. Electrostatic interactions were treated by Particle Mesh Ewald summation method [8] with cubic interpolation order of 4 and grid spacing of 1.6 Å with a real-space cutoff of 12 Å. All bonds in the enzyme and water were constrained by LINCS algorithm.

SUPPLEMENTARY FIGURES AND TABLES:

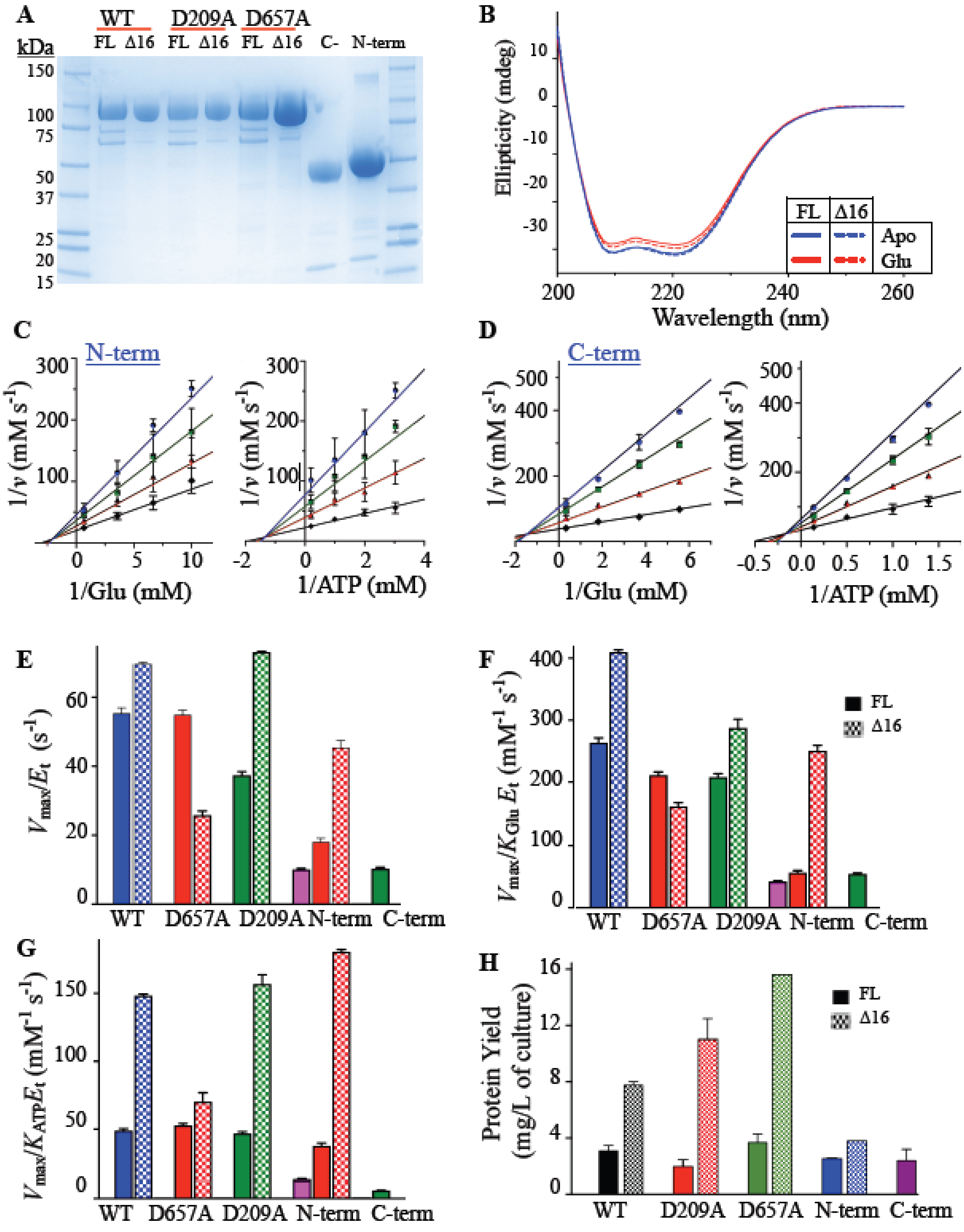

Supplementary Figure 1: Structural and catalytic characterization of human HK 2 variants. **(A)** SDS-PAGE analysis of the FL- and Δ16-HK2 variants of the WT, D209, and D657A as well as the separate N- and C-domains. Coomassie-stain was used to visualize the protein bands and ImagJ [18] was used to quantity purity >90%. **(B)** Far-UV CD spectra of the FL (solid line) and Δ16 (dashed line) variants of the WT-HK2 in the absence (blue) and presence of glucose (red). The CD spectra overlapped with minima at 208 and 222 nm. **(C-D)** IIIIIIIII **(E–G)** Kinetic parameters of the FL (solid bars) and Δ16 (checkered bars) variants of HK2 including different sizes of the N-domain including residues 1–469 (pink) and 1–479 (red). The rate was determined in the direction of formation of G6P. **(H)** Total protein yield of the FL- (solid bars) and Δ16- (checkered bars) HK2 variants per one liter of *E. coli* culture after completing all required purification steps.

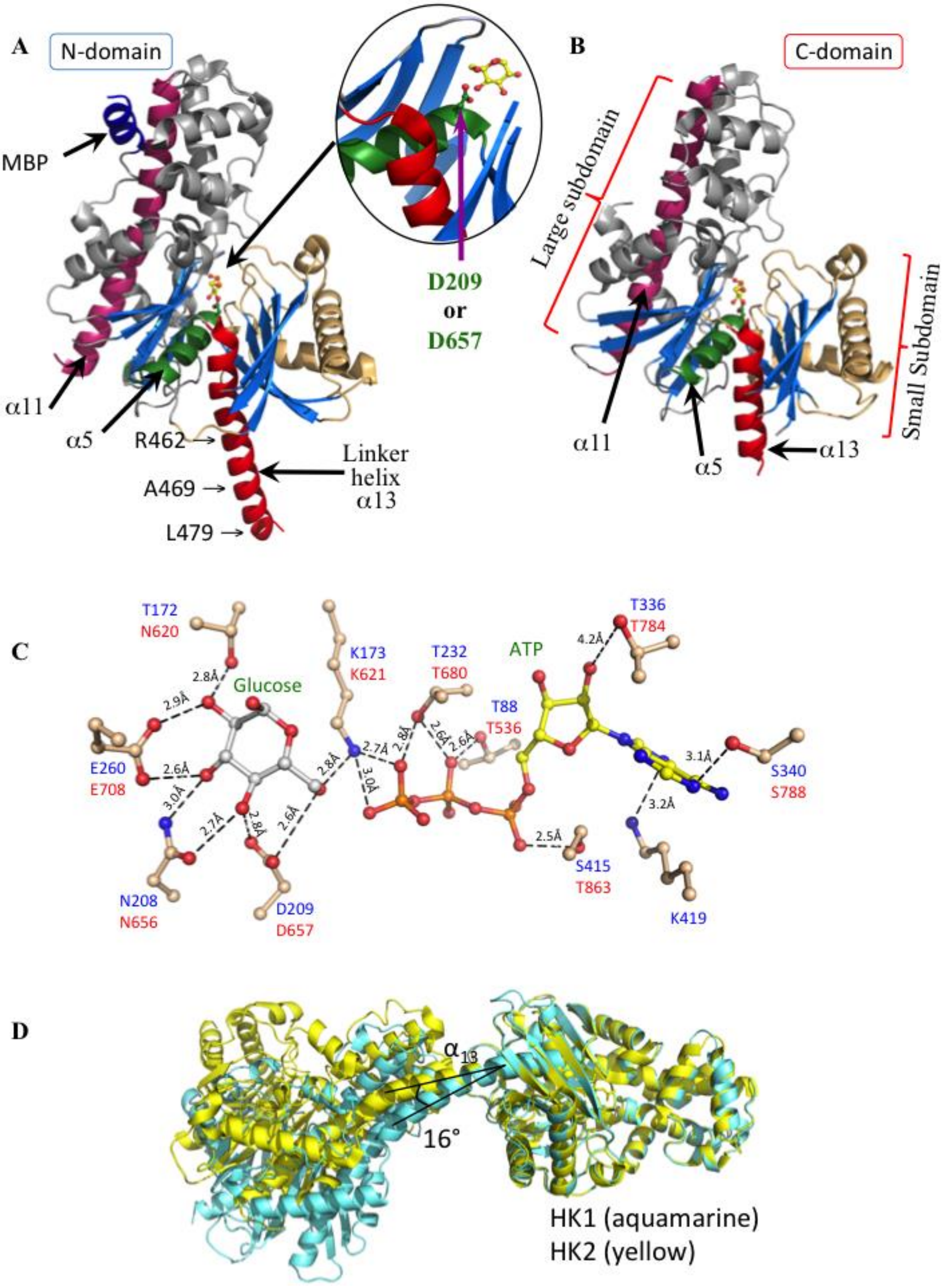
A
N-domain
MBP
α11
α5
R462
A469
L479
Linker
helix
α13
D209
or
D657
B
C-domain
Large subdomain
Small Subdomain
α11
α5
α13
C
T172
N620
Glucose
E260
E708
N208
N656
D209
D657
K173
K621
T232
T680
T88
T536
ATP
T336
T784
S340
S788
S415
T863
K419
2.8Å
2.9Å
2.6Å
3.0Å
2.7Å
2.8Å
2.6Å
2.8Å
2.7Å
3.0Å
2.8Å
2.6Å
2.6Å
4.2Å
3.1Å
3.2Å
2.5Å
D
α13
16°
HK1 (aquamarine)
HK2 (yellow)

Supplementary Figure 2: The Crystal Structure of Human HK2. **(A−B)** The N- and C-halves of HK2 with large (white) and small (gold) subdomains. The MBP of the N-half is dark blue. Helix α_{13} (red) protrudes out of the active site at the end of the N- and C-halves. Two 5 stranded β-sheets (blue) encloses the active site in addition to helices α_5 (green) and α_{13}. **Inset**: Helix α_5 that carries the catalytic residue D209 or D657 of the N- and C-halves, respectively, is perpendicular to helix α_{13}. **(C)** The glucose (white) and ATP (yellow) binding pockets in HK2 with the later modeled based on the crystal structure of HK4−ATP complex. **(D)** Overlay of the monomers of HK1 (aquamarine) and HK2 (yellow) in complex with glucose and G6P. Alignment was only possible on N-half but not the FL enzyme. Even though the structural folds of HK1 and HK2 domains are identical, a 16° bent on the linker helix of HK1 prevented its alignment to HK2. This figure was prepared using PyMol (Schrodinger LLC).

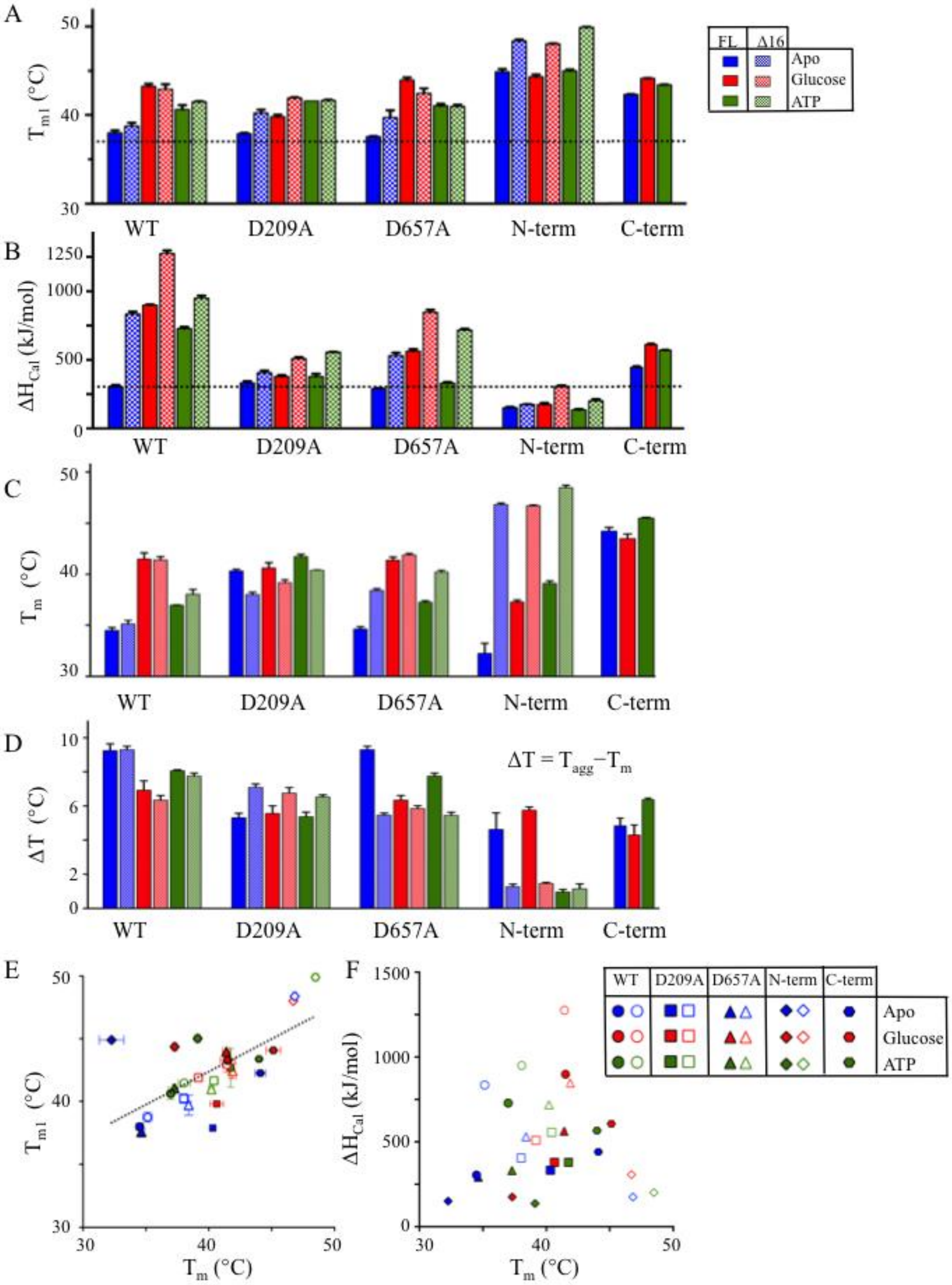
A
B
C
D
E
F
FL
Δ16
Apo
Glucose
ATP
T_{m1} (°C)
ΔH_{Cal} (kJ/mol)
T_m (°C)
ΔT (°C)
$\Delta T = T_{agg} - T_m$
WT
D209A
D657A
N-term
C-term

Supplementary Figure 3: Thermodynamic parameters of HK2 variants. **(A–B)** DSC parameters of FL (solid bars) and Δ16 (dotted bars) variants of the WT, D209, D657A, and N- and C-domains of HK2. T_{m1} was calculated from the temperature at the middle of the first transition, and ΔH_{cal} was determined from the area under the thermographic peaks of the DSC thermograms in figure 2. **(C−D)** DSF analysis of HK2 variants for the determination of T_m and T_{agg} in the presence of SYPRO Orange or Enzo ProteoStat reporter dyes, respectively. To confirm thermal unfolding preceded domain aggregation, ΔT ($T_{agg} - T_m$) was >5°C for all variants except the Δ16 variant of N-term due to its high T_m value. **(E−F)** Correlation of the T_m from DSF analysis against DSC parameters, ΔH_{cal} and T_{m1}, respectively. The FL (solid symbol) and Δ16 (open symbol) variants of the WT (●), D209A (■), and D657A (▲) mutants of HK2 are indicated in the background of N- (◆) and C-halves (⬣). Data are mean ± SD, n=3.

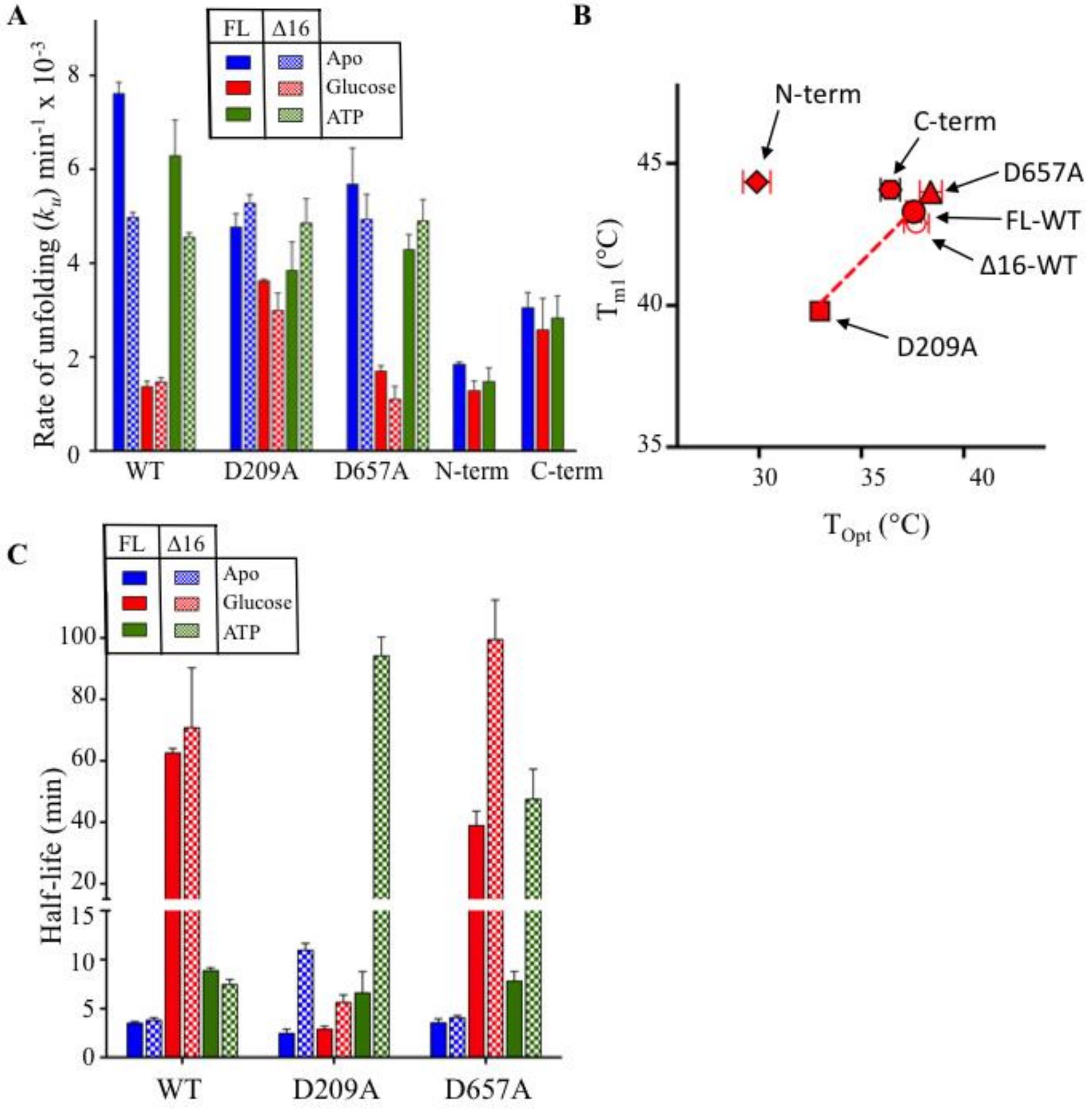

Supplementary Figure 4: Thermal Kinetic Stability, T_{Opt}, and Half-Life of HK2 variants. **(A)** The thermal rates of unfolding (k_u) of the HK2 variants FL (solid bars) and Δ16 (checkered bars). The k_u was measured after incubating HK2 at 37 °C in absence (blue) and presence of 5 mM Glucose (red) or 1 mM ATP (green) from the CD ellipticity readings at 222 nM. **(B)** Correlation between the optimum temperate of catalysis (T_{Opt}) and T_{m1} from the DSC analysis in the presence of 5 mM glucose. A direct correlation is observed for all HK2 variants with the lowest values recorded for D209A mutant except for the N-term did with very low T_{Opt} value in comparison to its high thermal stability, T_{m1}. **(C)** Half-life of HK2 variants measured by thermal inactivation kinetics. Symbols and colors are as in B. Data are mean ± SD, n=3.

Supplementary Table 1: HK2 variants.

Abbreviation	Configuration	Description	Range	Structural Conformation of HK2	MW* (kDa)
FL-HK2	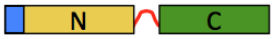	Full-length enzyme.	1 – 916	Cytosolic conformation.	102.4
Δ16-HK2	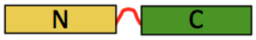	Deletion of mitochondrial binding peptide (MBP).	17 – 916	OMM bound conformation.	100.5
FL-N-term	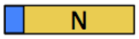	Full-length N-terminal domain.	1 – 479	Cytosolic conformation.	53.7
Δ16-N-term	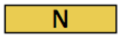	Deletion of MBP of N-terminal domain.	17 – 479	OMM bound conformation.	51.9
C-term	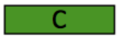	C-terminal domain.	465 – 916	Cytosolic conformation.	50.4

* The molecular weight (MW) was calculated using ExPasy Proteomics tools [19].

Supplementary Table 2: Crystallographic data and refinement statistics, related to Figure 1, and Supplementary Figure 2. Numbers in parentheses represent the highest resolution bin.

**PDB code**	2NZT
**Data collection**	
Space Group:	$P2_12_12_1$
Cell dimensions: *a, b, c* (Å)	94.9, 129.3, 187.2
Angles (°) α=β=γ	90
Resolution (Å)	25.0–2.45 (2.54-2.45)
Unique HKLs	85,698
Completeness (%)	98.2 (98.2)
R_{merge} (%)	8.2 (67)
Redundancy	5.5 (4.9)
**Refinement**	
Resolution (Å)	2.45
No. of atoms: protein/others	13,309/ 237
R_{work}/R_{free} (%)	23.0 / 28.7
B-factors: protein/others/Wilson ($Å^2$)	51.8
Average *B* factors ($Å^2$)	42.35
Protein: main chain/ side chain/ overall	50.56/51.49/51.00
Glucose & G6P	35.5/43.05
Water	39.17
R.m.s. deviations Bond lengths/angles	0.017Å / 1.427°
Ramachandran plot favored/outliners	95.75% (1640)/0.35% (6)

Supplementary Table 3: Kinetic parameters for HK2 variants were determined in the direction of formation of G6P at 25 °C and pH 7.5, related to Supplementary Figure 1.

	V/E_t (s^{-1})	K_{Glu} (mM)	K_{ATP} (mM)	$V/K_{Glu}E_t$ ($mM^{-1}\ s^{-1}$)	$V/K_{ATP}E_t$ ($mM^{-1}\ s^{-1}$)
FL (Residues: 1-916)	55.4 ± 1.5	0.21 ± 0.01	1.13 ± 0.08	264 ± 7	49 ± 1
Δ16 (Residues: 17-916)	69.7 ± 0.5	0.17 ± 0.01	0.47 ± 0.02	410 ± 3	148 ± 1
FL (D209A) (Residues: 1-916)	37.5 ± 1.0	0.18 ± 0.03	0.8 ± 0.08	208 ± 6	47 ± 1.3
Δ16 (D209A) (Residues: 17-916)	73.2 ± 0.2	0.26 ± 0.01	0.43 ± 0.06	289 ± 13	172 ± 27
FL (D657A) (Residues: 1-916)	55 ± 1.3	0.26 ± 0.02	1.04 ± 0.07	212 ± 5	53 ± 1
Δ16 (D657A) (Residues: 17-916)	25.9 ± 1.1	0.16 ± 0.01	0.37 ± 0.05	164 ± 5	70 ± 7
N-term (Residues: 1-469)	10.2 ± 0.2	0.24 ± 0.06	0.76 ± 0.12	42 ± 1	13 ± 1
N-term (Residues: 1-479)	18.1 ± 1.0	0.32 ± 0.06	0.48 ± 0.10	57 ± 3	38 ± 2
Δ16-N-term (Residues: 17-479)	45.5 ± 2.0	0.18 ± 0.01	0.25 ± 0.01	251 ± 8	180 ± 1
C-term (Residues: 465-916)	10.4 ± 0.3	0.19 ± 0.01	1.7 ± 0.2	55 ± 2	6.1 ± 0.2

SUPLEMENTARY REFERENCES:

1 Olender, R. and Elber, R. (1997) Yet another look at the steepest descent path. J Mol Struc-Theochem. **398**, 63-71

2 Kuettner, E. B., Kettner, K., Keim, A., Svergun, D. I., Volke, D., Singer, D., Hoffmann, R., Muller, E. C., Otto, A., Kriegel, T. M. and Strater, N. (2010) Crystal structure of hexokinase KlHxk1 of Kluyveromyces lactis: a molecular basis for understanding the control of yeast hexokinase functions via covalent modification and oligomerization. J Biol Chem. **285**, 41019-41033

3 Fischer, D. (2006) Servers for protein structure prediction. Curr Opin Struc Biol. **16**, 178-182

4 Sanbonmatsu, K. Y., Joseph, S. and Tung, C. S. (2005) Simulating movement of tRNA into the ribosome during decoding. P Natl Acad Sci USA. **102**, 15854-15859

5 Pronk, S., Pall, S., Schulz, R., Larsson, P., Bjelkmar, P., Apostolov, R., Shirts, M. R., Smith, J. C., Kasson, P. M., van der Spoel, D., Hess, B. and Lindahl, E. (2013) GROMACS 4.5: a high-throughput and highly parallel open source molecular simulation toolkit. Bioinformatics. **29**, 845-854

6 Whitford, P. C., Noel, J. K., Gosavi, S., Schug, A., Sanbonmatsu, K. Y. and Onuchic, J. N. (2009) An all-atom structure-based potential for proteins: Bridging minimal models with all-atom empirical forcefields. Proteins. **75**, 430-441

7 Ratje, A. H., Loerke, J., Mikolajka, A., Brunner, M., Hildebrand, P. W., Starosta, A. L., Donhofer, A., Connell, S. R., Fucini, P., Mielke, T., Whitford, P. C., Onuchic, J. N., Yu, Y. N., Sanbonmatsu, K. Y., Hartmann, R. K., Penczek, P. A., Wilson, D. N. and Spahn, C. M. T. (2010) Head swivel on the ribosome facilitates translocation by means of intra-subunit tRNA hybrid sites. Nature. **468**, 713-U143

8 Bussi, G., Donadio, D. and Parrinello, M. (2007) Canonical sampling through velocity rescaling. J Chem Phys. **126**

9 Elber, R., Roitberg, A., Simmerling, C., Goldstein, R., Li, H. Y., Verkhivker, G., Keasar, C., Zhang, J. and Ulitsky, A. (1995) Moil - a Program for Simulations of Macromolecules. Comput Phys Commun. **91**, 159-189

10 Kaminski, G. A., Friesner, R. A., Tirado-Rives, J. and Jorgensen, W. L. (2001) Evaluation and reparametrization of the OPLS-AA force field for proteins via comparison with accurate quantum chemical calculations on peptides. J Phys Chem B. **105**, 6474-6487

11 Kony, D., Damm, W., Stoll, S. and van Gunsteren, W. F. (2002) An improved OPLS-AA force field for carbohydrates. J Comput Chem. **23**, 1416-1429

12 Kirmizialtin, S., Nguyen, V., Johnson, K. A. and Elber, R. (2012) How Conformational Dynamics of DNA Polymerase Select Correct Substrates: Experiments and Simulations. Structure. **20**, 618-627

13 Pranata, J., Wierschke, S. G. and Jorgensen, W. L. (1991) Opls Potential Functions for Nucleotide Bases - Relative Association Constants of Hydrogen-Bonded Base-Pairs in Chloroform. J Am Chem Soc. **113**, 2810-2819

14 Berendsen, H. J. C., Grigera, J. R. and Straatsma, T. P. (1987) The Missing Term in Effective Pair Potentials. J Phys Chem-Us. **91**, 6269-6271

15 Smith, D. E. and Dang, L. X. (1994) Computer-Simulations of Nacl Association in Polarizable Water. J Chem Phys. **100**, 3757-3766

16 Allner, O., Nilsson, L. and Villa, A. (2012) Magnesium Ion-Water Coordination and Exchange in Biomolecular Simulations. J Chem Theory Comput. **8**, 1493-1502
17 Bussi, G., Donadio, D. and Parrinello, M. (2007) COMP 8-Canonical sampling through velocity rescaling. Abstr Pap Am Chem S. **234**
18 Schneider, C. A., Rasband, W. S. and Eliceiri, K. W. (2012) NIH Image to ImageJ: 25 years of image analysis. Nat Methods. **9**, 671-675
19 Gasteiger, E., Gattiker, A., Hoogland, C., Ivanyi, I., Appel, R. D. and Bairoch, A. (2003) ExPASy: The proteomics server for in-depth protein knowledge and analysis. Nucleic Acids Res. **31**, 3784-3788
